# RING-type E3 ligase RhSAREL promotes flower senescence by differentially regulating two TMBIM family members, RhTMBIM1 and RhTMBIM7, in *Rosa hybrida*

**DOI:** 10.1093/plphys/kiag587

**Published:** 2026-08-11

**Authors:** Jin Chen, Wentong Zhou, Siqi Zhao, Yubi Su, Yuhan Fan, Yuanyuan Wang, Jiaqi Zhao, Yiqing Liu, Feifei Yu, Changqing Zhang

**Affiliations:** Department of Ornamental Horticulture, College of Horticulture, China Agricultural University, Beijing 100193, China; Department of Ornamental Horticulture, College of Horticulture, China Agricultural University, Beijing 100193, China; Department of Ornamental Horticulture, College of Horticulture, China Agricultural University, Beijing 100193, China; Department of Ornamental Horticulture, College of Horticulture, China Agricultural University, Beijing 100193, China; Department of Ornamental Horticulture, College of Horticulture, China Agricultural University, Beijing 100193, China; Department of Ornamental Horticulture, College of Horticulture, China Agricultural University, Beijing 100193, China; Department of Ornamental Horticulture, College of Horticulture, China Agricultural University, Beijing 100193, China; Chongqing Key Laboratory for Germplasm Innovation of Special Aromatic Spice Plants, College of Smart Agriculture, Chongqing University of Arts and Sciences, Chongqing 402160, China; State Key Laboratory of Maize Bio-Breeding, College of Grassland Science and Technology, China Agricultural University, Beijing 100193, China; Department of Ornamental Horticulture, College of Horticulture, China Agricultural University, Beijing 100193, China

## Abstract

Flower senescence significantly impacts the postharvest quality of cut roses (*Rosa hybrida*). E3 ligases orchestrate a diverse array of processes in plants, including organ development and stress responses. However, the molecular mechanisms of E3 ligases in flower senescence remain largely unknown. Here, we report that *Senescence-associated RING-type E3 ligase* (*RhSAREL*), a RING-H2 E3 ligase gene, is highly expressed at the early stage of flower senescence. *RhSAREL*-RNAi transgenic rose plants showed increased flower longevity. We confirmed that RhSAREL interacts individually with 2 Transmembrane BAX Inhibitor-1 Motif-containing (TMBIM) family proteins, RhTMBIM1 and RhTMBIM7, both in vivo and in vitro. Both *RhTMBIM1* and *RhTMBIM7* showed stable transcript levels during flower senescence, while silencing *RhTMBIM1* or *RhTMBIM7* accelerated flower senescence. Biochemical assays revealed that RhSAREL ubiquitinates RhTMBIM7 and promotes its degradation via the 26S proteasome pathway, but not so for RhTMBIM1. Protein docking and interface analysis showed that RhSAREL blocks RhTMBIM1's calcium (Ca^2+^) leakage channel through its key N-terminal 26-amino-acid region. This interaction conformation disrupts RhTMBIM1-mediated Ca^2+^ homeostasis, thereby accelerating petal programed cell death and senescence. Taken together, the RING-H2 E3 ligase RhSAREL promotes rose flower senescence by differentially regulating RhTMBIM1 and RhTMBIM7 via E3 ligase activity-independent and -dependent pathways, respectively.

## Introduction

Organ senescence, the terminal phase of organ development, is not only prevalent in living organisms but also vital to their life activities. For example, floral senescence is often actively induced following pollination to remobilize nutrients and remove functionally redundant tissues (Liang et al. 2020; Sun et al. 2021b). Fruit senescence typically involves sugar accumulation, volatile emission, and tissue softening, which are crucial for attracting seed-dispersing organisms (Gapper et al. 2013). Leaf senescence serves as an adaptive strategy for plants to cope with adverse environments, primarily through the translocation of nutrients from leaves to more important regions, such as developing fruits and seeds (Guo and Gan 2005).

Organ senescence occurs in an orderly manner and generally culminates in programed cell death (PCD) (Gan 2018). This process is finely tuned by a multilayered regulatory network, including epigenetic, transcriptional, post-transcriptional, translational, and post-translational regulation (Thomas 2013; Guo et al. 2021; Miryeganeh 2022). DNA demethylase *DcROS1.1*, whose expression is downregulated by ethylene, has been reported to delay carnation flower senescence (Feng et al. 2024). By contrast, DNA demethylase *DML3* is induced during leaf senescence and promotes this process by activating a subset of senescence-associated genes (SAGs) in *Arabidopsis thaliana* (Yuan et al. 2020). In addition, transcription factors such as *WRKY53*, *MYC2*, and *NAP* positively regulate organ senescence (Miao et al. 2004; Qi et al. 2015; Zou et al. 2021), whereas *SOC1*, *HB6*, and *PLATZ9* negatively regulate it (Chen et al. 2017; Wu et al. 2017; Jiang et al. 2023). Flower senescence significantly affects the lifespan of ornamental plants, particularly cut roses, which is a key determinant of their commercial value. Given this, elucidating the regulatory mechanisms underlying flower senescence is essential to achieve precision breeding, allowing breeders to manipulate different types of regulators to meet diverse breeding objectives.

Ubiquitination is an important protein modification involved in post-translational regulation. It proceeds through a series of consecutive enzymatic reactions involving ubiquitin (Ub), Ub-activating enzyme (E1), Ub-conjugating enzyme (E2), Ub ligase (E3), and substrate proteins (Callis 2014). The most common outcome for ubiquitinated proteins is degradation via the 26S proteasome pathway (Swatek and Komander 2016). E3 ligases have attracted considerable attention because they can specifically recognize substrate proteins (Vierstra 2009). Several E3 ligases have been shown to modulate flower senescence. For instance, the Really Interesting New Gene (RING)-type E3 ligase *XA21 binding protein 3* (*XB3*), which is specifically expressed in senescing 4 o’clock (*Mirabilis jalapa*) flowers, promotes flower senescence (Xu et al. 2007). Furthermore, the Arabidopsis E3 ligase Coronatine Insensitive 1 (COI1) forms a protein complex with CONSTANS (CO) and jasmonate ZIM-domain 3 (JAZ3) to promote their degradation, thus releasing the repressor activity of JAZ3 on downstream JA-responsive factors during flower senescence (Serrano-Bueno et al. 2022). Ethylene antagonizes GA activity in the regulation of flower senescence by SCF-type E3 ligase SENESCENCE-ASSOCIATED F-BOX (RhSAF)-mediated ubiquitination and degradation of GIBBERELLIN INSENSITIVE DWARF1 (RhGID1), a GA receptor that delays petal senescence (Lu et al. 2024). In addition, numerous E3 ligases have been reported to be involved in leaf senescence (Guo et al. 2021; Wei et al. 2022; Ali et al. 2023; Tang et al. 2024a). However, the functions and underlying molecular mechanisms of E3 ligases in flower senescence are still poorly understood.

The Transmembrane BAX Inhibitor-1 Motif-containing (TMBIM) protein family appears to be ubiquitous, present in both eukaryotes and prokaryotes (Henke et al. 2011). TMBIM family proteins have at least 6 transmembrane helices and share a Bax1-I domain (PF01027). The first member of the TMBIM family, human TMBIM6 (also known as Bax inhibitor-1, BI-1), was initially identified as an inhibitor of Bax-induced cell death in yeast (Xu and Reed 1998). Most members of the TMBIM family have similar anti-apoptotic activities. For example, both human TMBIM1 and TMBIM2 inhibit Fas ligand (FasL)-induced apoptosis (Gubser et al. 2007; Shukla et al. 2011). Individual knockdown of *TMBIM2*, *TMBIM4*, or *TMBIM6* in different human cell lines leads to spontaneous apoptosis (Xu and Reed 1998; Fernández et al. 2007; Gubser et al. 2007). Elevated expression of mouse *TMBIM3* protects cells against endoplasmic reticulum (ER) stress-induced cell death (Rojas-Rivera et al. 2012). Moreover, the functions of TMBIM family proteins are conserved in plants. BI-1 orthologs from rice (*Oryza sativa*) and Arabidopsis suppress mammalian Bax-induced cell death (Kawai et al. 1999; Kawai-Yamada et al. 2001). Overexpression of *AtBI-1* enhances resistance to H_2_O_2_- and salicylic acid (SA)-induced cell death in tobacco (*Nicotiana tabacum*) suspension cells (Kawai-Yamada et al. 2004). AtBI-1 and AtLFG1/2/3 negatively regulate ER stress-induced cell death in Arabidopsis (Watanabe and Lam 2008; Guo et al. 2018; Wang et al. 2019). Accumulating evidence indicates that the effect of TMBIM family proteins on cell death is closely associated with the regulation of intracellular Ca^2+^ homeostasis (Ihara-Ohori et al. 2007; Bultynck et al. 2012; Liu 2017). Structural analyses of BsYetJ, a TMBIM family protein from *Bacillus subtilis*, revealed that it functions as a pH-sensitive calcium (Ca^2+^)-leak channel (Chang et al. 2014). Notably, 2 aspartate residues serving as pH sensors are highly conserved across the TMBIM family (Chang et al. 2014). All human TMBIM family proteins can reduce Ca^2+^ levels in the endoplasmic reticulum, leading to increased resistance to cell death (Nakamura et al. 2000; Lisak et al. 2015). AtBI-1 delays methyl jasmonate (MeJA)-induced leaf senescence by suppressing the elevation of [Ca^2+^]_cyt_ (Yue et al. 2012). However, the potential roles of TMBIM family proteins in flower senescence remain to be elucidated.

In this study, we found that the senescence- and ethylene-induced RING-H2 E3 ligase, RhSAREL, promotes rose flower senescence. RhSAREL interacts with RhTMBIM1 and RhTMBIM7 individually, both of which delay rose flower senescence. RhSAREL ubiquitinates RhTMBIM7 for degradation via the 26S proteasome pathway. In addition, the interaction between RhSAREL and RhTMBIM1 disrupts the RhTMBIM1-mediated Ca^2+^ homeostasis and accelerates flower senescence. These results reveal the differential regulation of 2 TMBIM family proteins by RhSAREL and provide new insights into the molecular mechanisms of flower senescence.

## Results

### RhSAREL is a senescence-associated E3 ligase and is localized to the endoplasmic reticulum

**Figure 1:**
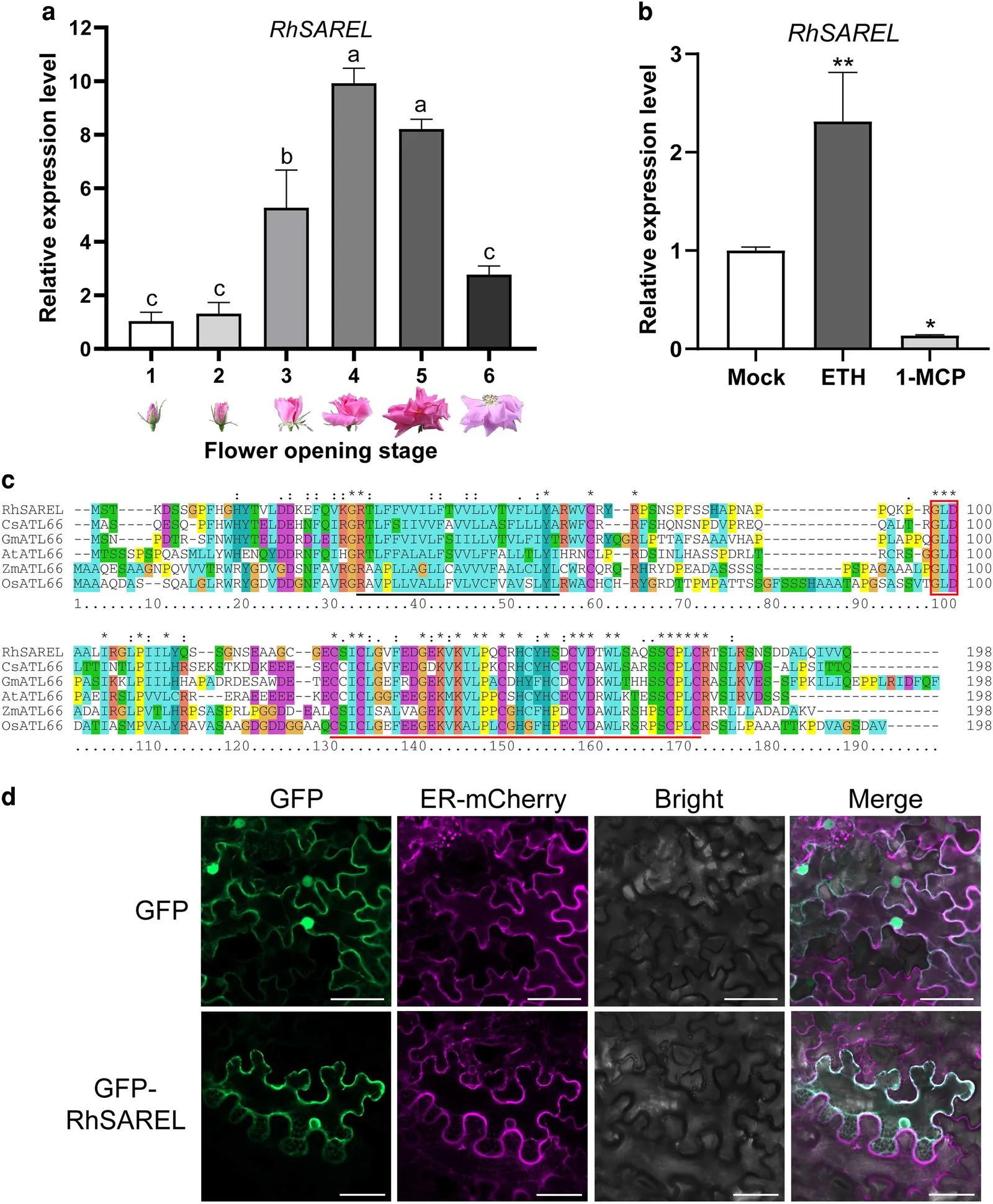
RhSAREL is a senescence-associated E3 ligase and localized to ER. a) Expression of *RhSAREL* in petals at different flower opening stages detected by RT-qPCR. Data are shown as means ± standard deviation (SD) (n = 3). Different letters indicate statistically significant differences (*P* < 0.05) analyzed by one-way ANOVA with Tukey's test. b) Expression of *RhSAREL* in rose flowers treated with air (Mock), 10 μL/L ethylene (ETH) or 2 μL/L 1-MCP for 24 h. *RhUBI2* was used as a reference gene. Data are shown as means ± SD (n = 3). Asterisks indicate statistically significant differences according to one-way ANOVA with Dunnett's test (*P < 0.05, **P < 0.01). c) Sequence alignment of RhSAREL and its homolog from other species. The first line (black) and the second line (red) indicate the transmembrane region and the RING-H2 domain, respectively. The box indicates the conserved GLD motif in ATL proteins. The protein sequences used are listed below: RhSAREL, *Rosa hybrida* XP_024164126.1; CsATL66, *Citrus sinensis* XP_052295277.1; GmATL66, *Glycine max* XP_003538474.1; AtATL66, *Arabidopsis thaliana* NP_187722.1; ZmATL66, *Zea mays* XP_008656384.1; OsATL66, *Oryza sativa* XP_015618570.1. d) Subcellular localization of RhSAREL in epidermal cells of tobacco leaves. The ER-rk *CD3-959* vector was used as the endoplasmic reticulum (ER) marker (Nelson et al. 2007). *Agrobacterium tumefaciens* (GV3101) carrying *pSuper::GFP-RhSAREL* or *pSuper::GFP* and a marker vector were co-infiltrated into tobacco leaves. Fluorescence signals were imaged using a confocal laser-scanning microscope (Zeiss, Germany) 72 h after infiltration. Bars, 50 μm.

By analyzing previous transcriptome data of rose petals (Liu et al. 2025), we identified a putative RING-type E3 ligase gene, whose fragments per kilobase of transcript per million mapped reads (FPKM) value was continuously elevated during flower senescence. We named this gene *RhSAREL* (*Senescence-associated RING-type E3 Ligase*) for further investigation. We confirmed the expression profile of *RhSAREL* using reverse transcription-quantitative PCR (RT-qPCR). The expression of *RhSAREL* remained at a low level at the first 2 stages and dramatically increased by approximately ten-fold at stage 4 (Fig. 1a). Given the importance of ethylene signaling in flower senescence (Ma et al. 2018), we analyzed the expression pattern of *RhSAREL* in petals after ethylene treatment. *RhSAREL* was induced by ethylene and significantly repressed by 1-methylcyclopropene (1-MCP), a competitive ethylene inhibitor (Fig. 1b).

Phylogenetic analysis revealed that RhSAREL is homologous to Arabidopsis (*A. thaliana*) ATL66 (Fig. S1a). In addition to the RING-H2 domain, RhSAREL and its orthologs harbor a transmembrane domain and a GLD motif, which are conserved in all Arabidopsis Tóxicos en Levadura (ATL) proteins (Serrano et al. 2006; Aguilar-Hernández et al. 2011) (Fig. 1c and Fig. S1b). Moreover, to investigate the expression patterns of other RING-H2 E3 ligase genes in the transcriptome data, we first identified 255 RING-H2 E3 ligase members in the rose genome based on the consensus sequence of the RING-H2 domain (C-X_2_-C-X_9-39_-C-X_1-3_-H-X_2-3_-H-X_2_-C-X_4-48_-C-X_2_-C) (Stone et al. 2005) (Fig. S2a). A total of 54 genes were characterized as differentially expressed during flower senescence, based on the criteria of a mean FPKM (across stages 2 to 5) > 10, a fold change (stage 5 vs. stage 2) > 1.5 or <0.67, and FDR < 0.05. Among these, 30 were upregulated, with *RhSAREL* having the highest fold change of 21.25 (S5 vs. S2), and 24 were downregulated (Fig. S2b).

We then examined the subcellular localization of RhSAREL by transiently co-expressing GFP-RhSAREL with the endoplasmic reticulum (ER) marker ER-mCherry in tobacco (*Nicotiana benthamiana*) leaves (Nelson et al. 2007). The green fluorescence signals of GFP-RhSAREL overlapped with the red fluorescence signals of ER-mCherry, indicating that GFP-RhSAREL localizes to the ER (Fig. 1d).

Since RhSAREL is a putative E3 ligase, we attempted to test the E3 ligase activity of RhSAREL by in vitro ubiquitination assays. In the presence of His-tagged AtUBA2 (E1-His), His-tagged AtUBC10 (E2-His), and ubiquitin, a smear band was detected by anti-Ub antibody, suggesting that RhSAREL is an active E3 ligase. Moreover, the self-ubiquitinated form of RhSAREL was abolished when 3 key amino acids (aa) residues (Cys^123^, His^125^, His^128^) of the RING-H2 domain were mutated to alanine (Stone et al. 2005) (Fig. S3). This result demonstrates that the E3 ligase activity of RhSAREL is dependent on the RING-H2 domain.

### RhSAREL accelerates rose petal senescence

To explore the function of *RhSAREL* in petal senescence, we performed virus-induced gene silencing (VIGS) assays in rose plantlets. A 400 bp fragment of the *RhSAREL* gene was constructed in the pTRV2 vector, which was subsequently introduced into rose buds using Agrobacterium. RT-qPCR analysis confirmed that the expression of *RhSAREL* in silenced petals was approximately twofold lower than that in control petals (Fig. S4c). Flower longevity of infected buds from stage 5 was recorded as previously reported (Lu et al. 2024). Compared to the control flowers, *RhSAREL*-silenced flowers exhibited delayed petal wilting and abscission (Fig. S4a). Silencing of *RhSAREL* prolonged flower longevity by approximately 2 d relative to that of the TRV control (Fig. S4b). In line with the flower phenotype, *RhSAG12,* a senescence marker gene (Lu et al. 2019), was significantly repressed in *RhSAREL*-silenced petals compared to control petals (Fig. S4d), indicating a slower senescence process. In addition, trypan blue staining revealed that silencing *RhSAREL* led to milder cell death than that in the control (Fig. S5a). Electrolyte leakage, an indicator of plant cell death induced by senescence, was alleviated in petal discs from *RhSAREL*-silenced plants (Fig. S5c). Finally, we detected the transcript levels of PCD-associated genes, including *RhBFN1*, *RhPASPA3*, and *RhEXI1* (Olvera-Carrillo et al. 2015). RT-qPCR results showed that these 3 genes were significantly downregulated in *RhSAREL*-silenced petals (Fig. S5e).

**Figure 2:**
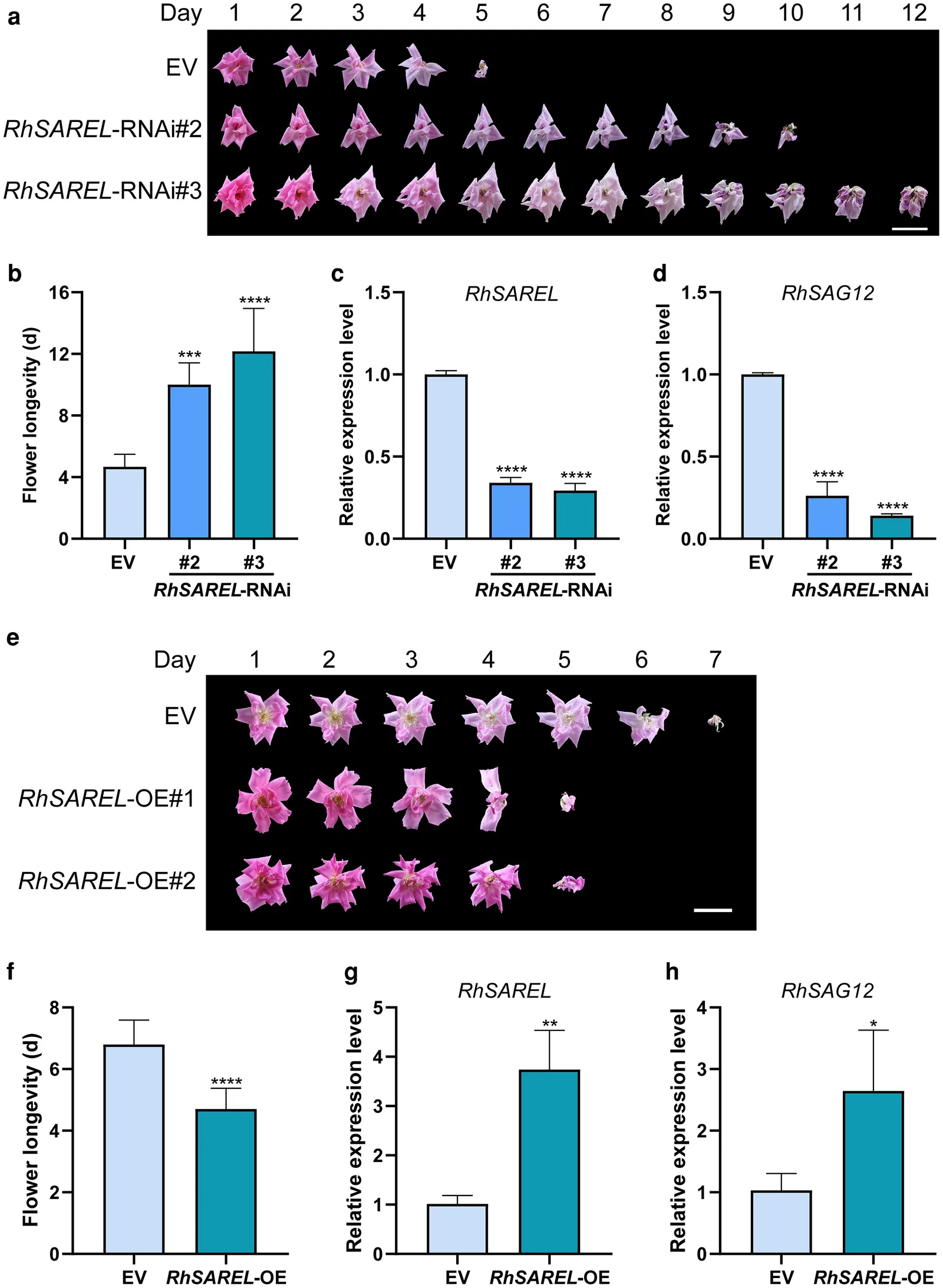
*RhSAREL* positively regulates petal senescence. a) Senescence process of empty vector (EV) and *RhSAREL*-RNAi transgenic rose flowers. Flowers were photographed daily from stage 5 until complete senescence. Bars, 5 cm. b) Flower longevity of EV and *RhSAREL*-RNAi rose flowers. Data are shown as means ± SD (n = 6). c) Expression of *RhSAREL* in EV and *RhSAREL*-RNAi petals. d) Expression of senescence marker gene *RhSAG12* in EV and *RhSAREL*-RNAi petals. e) Senescence process of control and *RhSAREL* overexpression rose flowers. Bars, 5 cm. f) Flower longevity of control and *RhSAREL* overexpression rose flowers. Data are shown as means ± SD (n = 10). g) Expression of *RhSAREL* in control and *RhSAREL* overexpression petals. h) *RhSAG12* expression in control and *RhSAREL* overexpression petals. The outermost petal of each flower at stage 5 was sampled for RNA extraction and expression analysis. *RhUBI2* was used as a reference gene. Data are shown as means ± SD, n = 3 in c), d), g), and h). For b) to d), asterisks indicate statistically significant differences according to one-way ANOVA with Dunnett's test (***P < 0.001, ****P < 0.0001). For f) to h), asterisks indicate statistically significant differences according to *t*-test (*P < 0.05, **P < 0.01, ****P < 0.0001).

We further generated *RhSAREL*-RNAi transgenic rose lines by *Agrobacterium tumefaciens*-mediated transformation of somatic embryos for subsequent phenotypic analysis. We observed significantly reduced transcript levels of *RhSAREL* and *RhSAG12* in the petals of the *RhSAREL*-RNAi lines (Fig. 2c and d). Like *RhSAREL*-silenced plants in VIGS assays, *RhSAREL*-RNAi transgenic plants displayed delayed flower senescence (Fig. 2a and b).

We transiently overexpressed *RhSAREL* in rose flowers using Agrobacterium infection. RT-qPCR analysis revealed an almost 4-fold increase in *RhSAREL* expression in *RhSAREL*-overexpressing petals relative to that in control petals (Fig. 2g). Overexpression of *RhSAREL* in flowers led to a substantial reduction in flower longevity of approximately 30% compared to the vector control (Fig. 2e and f). In petals with *RhSAREL* overexpression, the expression of *RhSAG12* was significantly higher than that in control petals (Fig. 2h). Consistent with this, increased cell death was observed in the petals overexpressing *RhSAREL* (Fig. S5b). Additionally, *RhSAREL* overexpression promoted electrolyte leakage from senescent petal discs (Fig. S5d). Furthermore, 3 PCD-associated genes were significantly upregulated in *RhSAREL*-overexpressing petals (Fig. S5f). These results demonstrate that *RhSAREL* plays a positive role in rose petal senescence.

### RhSAREL interacts with RhTMBIM1 and RhTMBIM7 in vivo and in vitro

To elucidate the molecular mechanism by which RhSAREL is involved in petal senescence, a cDNA library of rose petals was used to screen for potential RhSAREL-interacting proteins using yeast two-hybrid (Y2H) screening. We obtained 57 putative interactors from 64 sequenced positive clones (Table S1). Among these candidates, we identified the gene *BI-1* (*RchiOBHm_Chr7g0205291*) and its homolog *BI-1-like* (*RchiOBHm_Chr4g0419241*). Both of them belong to the Transmembrane BAX Inhibitor-1 Motif-containing (TMBIM) protein family, which is a highly conserved cell death suppressor family in eukaryotes (Rojas-Rivera and Hetz 2015). Phylogenetic analysis identified 8 rose TMBIM family members, designated as *RhTMBIM1-8* based on their phylogenetic distribution, where *RchiOBHm_Chr7g0205291* and *RchiOBHm_Chr4g0419241* correspond to *RhTMBIM1* and *RhTMBIM7*, respectively (Fig. S6).

**Figure 3:**
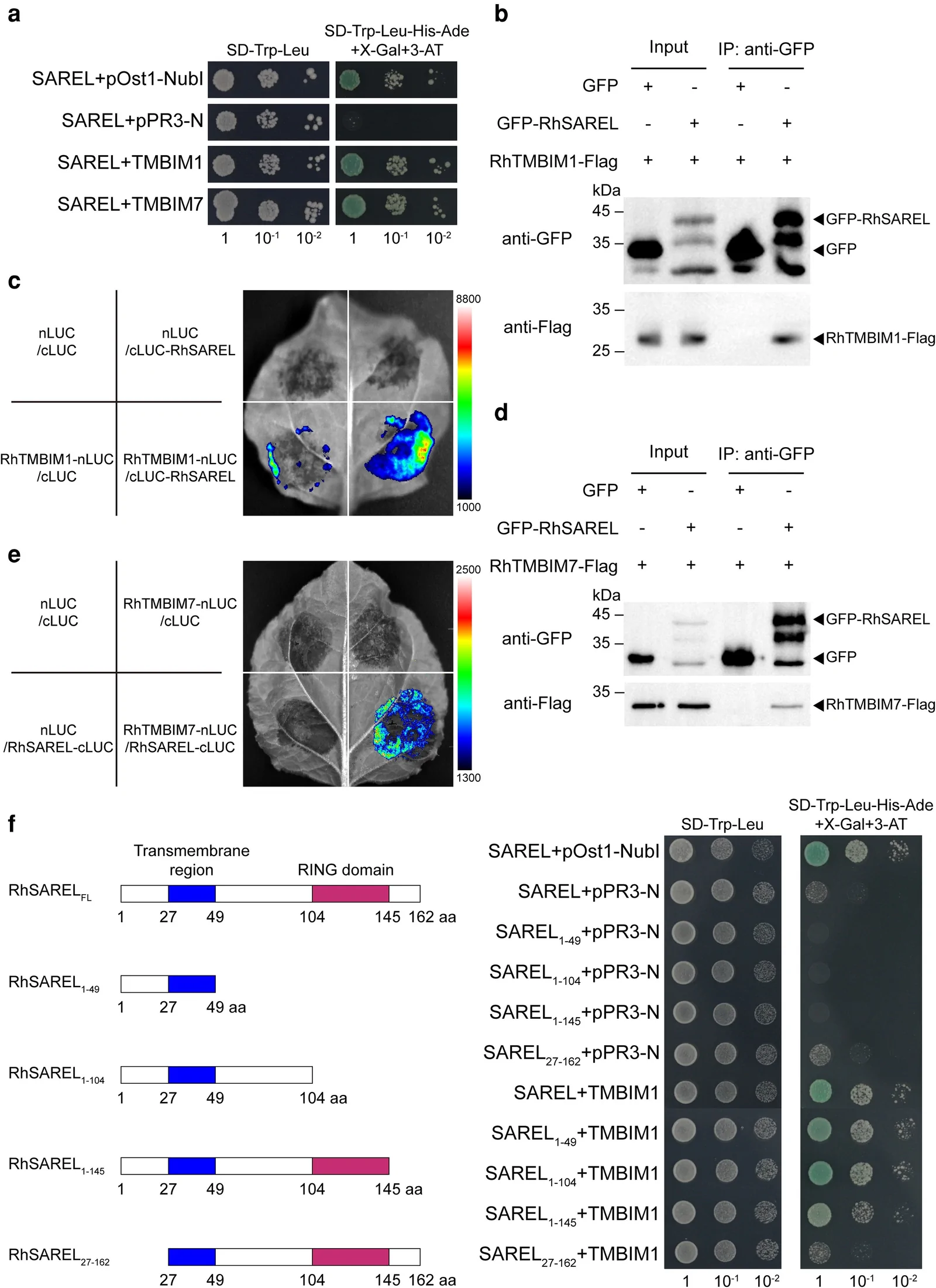
RhSAREL interacts with RhTMBIM1 and RhTMBIM7 individually. a) Interaction of RhSAREL with RhTMBIM1 or RhTMBIM7 in Y2H assays. Transformed yeast cells were serially diluted and cultured on SD-Trp-Leu medium as well as SD-Trp-Leu-His-Ade medium supplemented with 40 μg/mL X-Gal and 5 mM 3-AT. Combinations of pBT3-STE-RhSAREL with pOst1-NubI (positive control) and empty pPR3-N (negative control) vectors were used. b) Interaction of RhSAREL with RhTMBIM1 in Co-IP assays. The GFP-RhSAREL or GFP was co-infiltrated with RhTMBIM1-Flag into tobacco leaves. After 3 d of infiltration, the protein extracts were immunoprecipitated with GFP-Trap magnetic beads. The precipitates were analyzed using anti-GFP and anti-Flag antibodies. c) Interaction of RhSAREL with RhTMBIM1 in SLC assays. The combinations of nLUC/cLUC, nLUC/cLUC-RhSAREL, and RhTMBIM1-nLUC/cLUC served as negative controls. The luciferase activity was analyzed on the third day after infiltration. The pseudocolor indicates the intensity of luminescence. d) Interaction of RhSAREL with RhTMBIM7 in Co-IP assays. The GFP-RhSAREL or GFP was co-expressed with RhTMBIM7-Flag in tobacco leaves. The protein extracts were precipitated using GFP-Trap magnetic beads, and immunoblotting analysis was conducted with anti-GFP and anti-Flag antibodies. e) Interaction of RhSAREL with RhTMBIM7 in SLC assays. The combinations of nLUC/cLUC, nLUC/RhSAREL-cLUC, and RhTMBIM7-nLUC/cLUC served as negative controls. f) Interaction of RhSAREL or its truncated variants with RhTMBIM1 in Y2H assays. The domains of RhSAREL were analyzed by SMART (https://smart.embl.de/). Transformed yeast cells were subjected to serial dilutions and grown on SD-Trp-Leu medium and SD-Trp-Leu-His-Ade medium with 40 μg/mL X-Gal and 10 mM 3-AT. The combination of pBT3-STE-RhSAREL and pOst1-NubI vectors was employed as a positive control. Combinations of pBT3-STE-RhSAREL or its truncated variants with empty pPR3-N vectors were used as negative controls.

We then cloned the full-length coding sequences (CDS) of *RhTMBIM1* and *RhTMBIM7* into the prey vector pPR3-N and further validated their interaction with RhSAREL in the DUAL membrane system (Fig. 3a). Next, we performed co-immunoprecipitation (Co-IP) and split-luciferase complementation (SLC) assays to examine whether RhSAREL interacts with RhTMBIM1 or RhTMBIM7 in vivo. In the Co-IP assays, total protein was extracted from tobacco leaves co-expressing GFP-RhSAREL with RhTMBIM1-Flag or RhTMBIM7-Flag. After immunoprecipitation with anti-GFP magnetic beads, RhTMBIM1-Flag and RhTMBIM7-Flag were detected using anti-Flag antibodies in samples co-expressing GFP-RhSAREL and RhTMBIM1-Flag or GFP-RhSAREL and RhTMBIM7-Flag, respectively, but not in the GFP controls (Fig. 3b and d). In the SLC assays, stronger luminescence signals were observed in the regions of tobacco leaves co-infiltrated with *cLUC-RhSAREL*/*RhTMBIM1-nLUC* and *RhSAREL-cLUC*/*RhTMBIM7-nLUC*, compared with those in the negative control regions (Fig. 3c and e). These results indicate that RhSAREL interacts with RhTMBIM1 and RhTMBIM7 both in vitro and in vivo.

To determine which domain of RhSAREL is responsible for its interaction with TMBIM family proteins, we chose RhTMBIM1 as the prey protein and generated 4 truncated RhSAREL variants, which were cloned into the bait vector pBT3-STE. The Y2H assay revealed that deletion of the N-terminal 26-aa region of RhSAREL completely abolished its interaction with RhTMBIM1, while separate deletion of the C-terminus, RING domain, or GLD motif of RhSAREL had no such effect. Moreover, a truncated variant of RhSAREL (SAREL_1-49_) consisting of its N-terminus and the transmembrane region still retained this interaction (Fig. 3f). The Co-IP assay demonstrated that RhTMBIM1 did not co-precipitate with RhSARELΔN (lacking the N-terminal 26-aa) (Fig. S7a). To further confirm this, we performed the SLC assays and found that no luminescence signal was detected in the regions of tobacco leaves co-infiltrated with *cLUC-RhSARELΔN*/*RhTMBIM1-nLUC* (Fig. S7b). These results indicate that the N-terminal 26-aa region of RhSAREL is critical for its interaction with RhTMBIM1. We further investigated the domains of RhTMBIM1 mediating its interaction with RhSAREL by Y2H assays. However, no specific domains of RhTMBIM1 were identified that could individually mediate this interaction (Fig. S7c).

### RhSAREL ubiquitinates RhTMBIM7 for degradation

To assess whether TMBIM family members respond to petal senescence, we analyzed their expression profiles using transcriptome data. *RhTMBIM1* and *RhTMBIM7* were the main family members expressed during petal senescence with relatively stable expression levels (Table S2, Fig. S8). These results suggest that RhTMBIM1 and RhTMBIM7 may be regulated by post-translational modifications.

**Figure 4:**
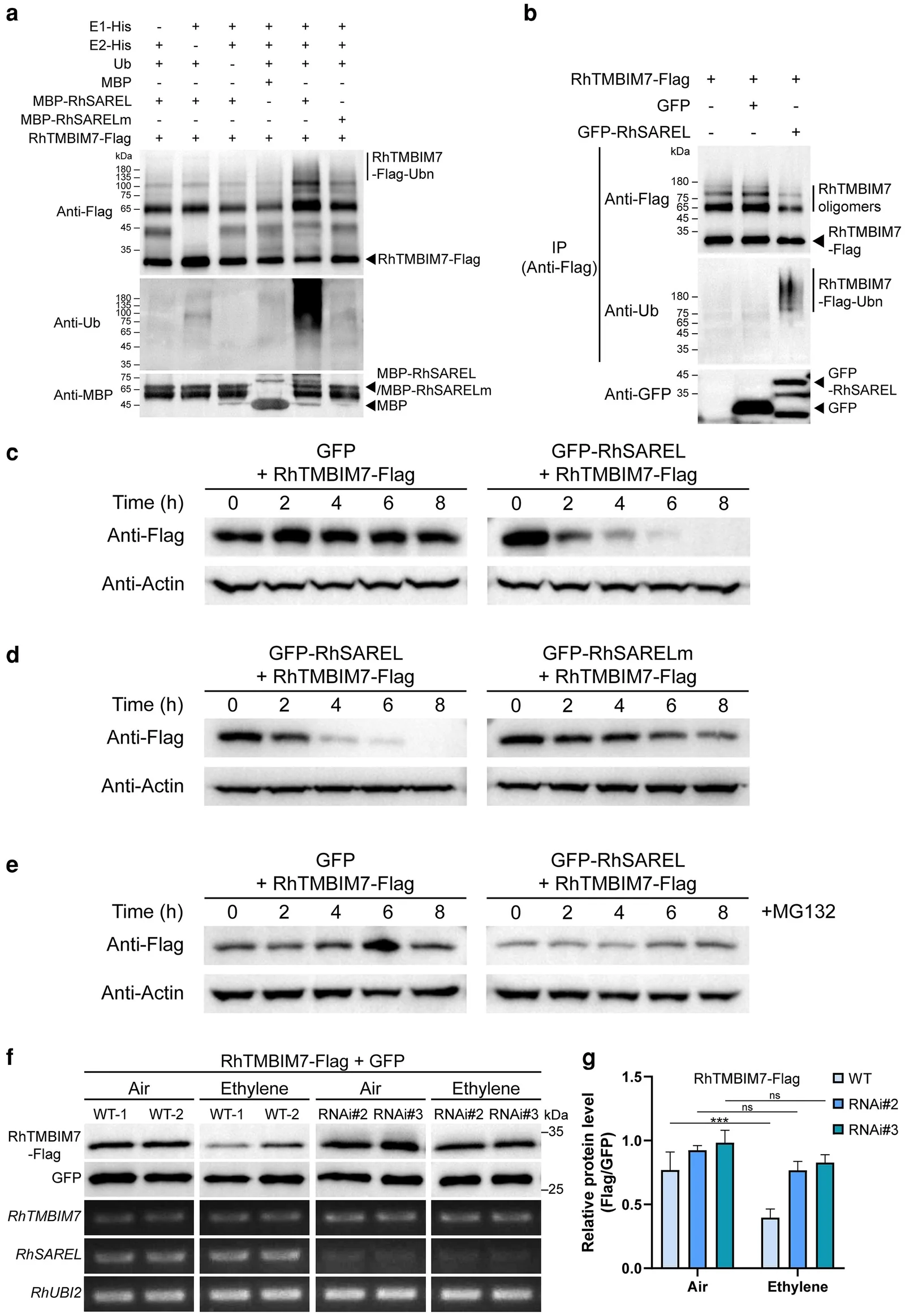
RhSAREL ubiquitinates RhTMBIM7 and promotes its degradation via the 26S proteasome pathway. a) RhSAREL ubiquitinates RhTMBIM7 in vitro. MBP and MBP-RhSARELm served as negative controls. The immunoprecipitated RhTMBIM7-Flag from tobacco leaves was used as the substrate. Immunoblot analysis was performed using anti-Flag, anti-Ub, and anti-MBP antibodies. RhTMBIM7-Flag-Ubn represents the ubiquitinated RhTMBIM7-Flag protein bands. b) RhSAREL ubiquitinates RhTMBIM7 in vivo. RhTMBIM7-Flag was transiently co-expressed with GFP or GFP-RhSAREL in tobacco leaves for 3 d. The leaves were treated with 50 μM MG132 12 h before harvest. Total proteins were extracted and immunoprecipitated using anti-Flag magnetic beads. The ubiquitination of RhTMBIM7-Flag was detected by the anti-Ub antibody. c) RhSAREL promotes the degradation of RhTMBIM7 in semi-in vivo protein degradation assays. The protein extract from tobacco leaves expressing RhTMBIM7-Flag was mixed with the protein extract from leaves expressing GFP or GFP-RhSAREL and incubated at 4°C. The abundance of RhTMBIM7-Flag protein at different time points was determined by immunoblotting using an anti-Flag antibody. Actin was used as an internal control. d) RhSARELm has no effect on the degradation of RhTMBIM7 in semi-in vivo protein degradation assays. The protein extract from leaves expressing RhTMBIM7-Flag was co-incubated with that from leaves expressing GFP-RhSAREL or GFP-RhSARELm at 4°C. e) MG132 treatment inhibits the degradation of RhTMBIM7 in semi-in vivo protein degradation assays. The mixtures containing different combinations were supplemented with 50 μM MG132. f) Ethylene promotes the *RhSAREL*-dependent degradation of RhTMBIM7 in rose petals. RhTMBIM7-Flag was transiently overexpressed in WT or *RhSAREL*-RNAi petals for 72 h. The infected petals were exposed to air or 10 μL/L ethylene for 24 h prior to harvest. The abundance of RhTMBIM7-Flag protein was assessed by immunoblotting using an anti-Flag antibody. GFP was used as an internal control. Transcript levels of *RhTMBIM7* and *RhSAREL* were analyzed by RT-PCR with *RhUBI2* as the internal reference. g) Quantification of RhTMBIM7-Flag protein level in f). The signal intensity of protein bands was quantified with ImageJ software. The relative protein level of RhTMBIM7-Flag was normalized to that of GFP. Data are shown as means ± SD (n = 3). Asterisks indicate statistically significant differences as determined by two-way ANOVA (***P < 0.001). All the experiments were biologically repeated 3 times with similar results.

Given that RhSAREL is an active E3 ligase, we first investigated whether RhSAREL ubiquitinates RhTMBIM1 and RhTMBIM7. We transiently expressed RhTMBIM1-Flag and RhTMBIM7-Flag in tobacco leaves. The immunoprecipitated proteins were used as substrates for in vitro ubiquitination assays. We observed an increased abundance of ubiquitinated RhTMBIM7-Flag upon addition of MBP-RhSAREL to the reaction, whereas the inactive variant MBP-RhSARELm had no effect (Fig. 4a and Fig. S9). However, similar smear bands of RhTMBIM1-Flag were detected in both the MBP-RhSAREL co-incubation and control groups, indicating that MBP-RhSAREL could not ubiquitinate RhTMBIM1-Flag in vitro (Fig. S10a).

We also performed in vivo ubiquitination assays. RhTMBIM7-Flag was coexpressed with GFP-RhSAREL in tobacco leaves, and samples with sufficiently expressed proteins were subjected to immunoprecipitation by anti-Flag magnetic beads. The ubiquitination level of RhTMBIM7-Flag was significantly higher than when RhTMBIM7-Flag was expressed alone or coexpressed with *GFP* (Fig. 4b). There was no significant change in the ubiquitination level of RhTMBIM1-Flag when RhTMBIM1-Flag was co-expressed with GFP-RhSAREL (Fig. S10b). These results collectively indicate that RhTMBIM7 is a ubiquitination target of RhSAREL, while RhTMBIM1 is not.

To examine whether RhSAREL regulates the degradation of RhTMBIM1 and RhTMBIM7, we individually expressed GFP-RhSAREL, RhTMBIM1-Flag, and RhTMBIM7-Flag in tobacco leaves for semi-in vivo degradation assays. Total protein was extracted from each sample and then mixed with protein extracts containing RhSAREL. The reaction mixtures were collected at various time points. RhTMBIM7-Flag degradation was accelerated upon incubation with total protein extract containing GFP-RhSAREL, whereas RhTMBIM1-Flag protein levels were not affected (Fig. 4c and Fig. S10c). Then, we co-incubated protein extracts from leaves expressing RhTMBIM7-Flag with those expressing GFP-RhSARELm (E3 ligase activity-deficient mutant). We found that incubation with GFP-RhSARELm inhibited the degradation of RhTMBIM7-Flag, indicating that this degradation was dependent on the E3 ligase activity of RhSAREL (Fig. 4d). RhTMBIM7-Flag protein remained stable when MG132, a proteasome inhibitor, was added to the reaction (Fig. 4e). Furthermore, we tested whether the ethylene signal promotes the degradation of RhTMBIM7. We transiently co-expressed RhTMBIM7-Flag and GFP in petals of *RhSAREL*-RNAi transgenic rose plants. Ethylene treatment was applied 2 d after infiltration, and the protein level of RhTMBIM7-Flag was detected 24 h after treatment. The results show that ethylene treatment did not change the *RhTMBIM7* transcript level; however, it significantly decreased the protein abundance of RhTMBIM7-Flag in WT petals, whereas there were no obvious changes in the *RhSAREL*-RNAi petals (Fig. 4f and g). These results demonstrate that ethylene-induced RhSAREL ubiquitinates RhTMBIM7 to promote its degradation via the 26S proteasome pathway but does not do so for RhTMBIM1.

To investigate the role of *RhTMBIM7* in petal senescence, we performed VIGS assays to silence *RhTMBIM7* in rose flowers. RT-qPCR analysis confirmed the reduced expression of *RhTMBIM7* in silenced petals (Fig. S11c). Silencing of *RhTMBIM7* resulted in significantly accelerated senescence (Fig. S11a and b). In silenced petals, the senescence marker gene *RhSAG12* showed a higher transcript level than that in control petals (Fig. S11d). These results suggest that *RhTMBIM7* acts as a senescence suppressor.

To further verify whether elevated RhTMBIM7 protein levels underlie the delayed senescence observed in *RhSAREL*-RNAi petals, we silenced both *RhSAREL* and *RhTMBIM7* in rose flowers by inserting specific fragments of these 2 genes in tandem into the pTRV2 vector. Compared to the control petals, TRV-*RhSAREL*/*RhTMBIM7* petals showed significantly downregulated expression levels of *RhSAREL* and *RhTMBIM7* (Fig. S12c and d). Silencing *RhSAREL* alone caused prolonged flower longevity, as mentioned in the previous section, while silencing both *RhSAREL* and *RhTMBIM7* resulted in a senescence phenotype comparable to that of the TRV control (Fig. S12a and b). The expression of *RhSAG12* in TRV-*RhSAREL*/*RhTMBIM7* petals was not significantly different from that in TRV control petals (Fig. S12e). These results suggest that RhSAREL accelerates rose flower senescence partially by promoting the degradation of RhTMBIM7.

### RhSAREL disrupts RhTMBIM1-mediated Ca^2+^ homeostasis

**Figure 5:**
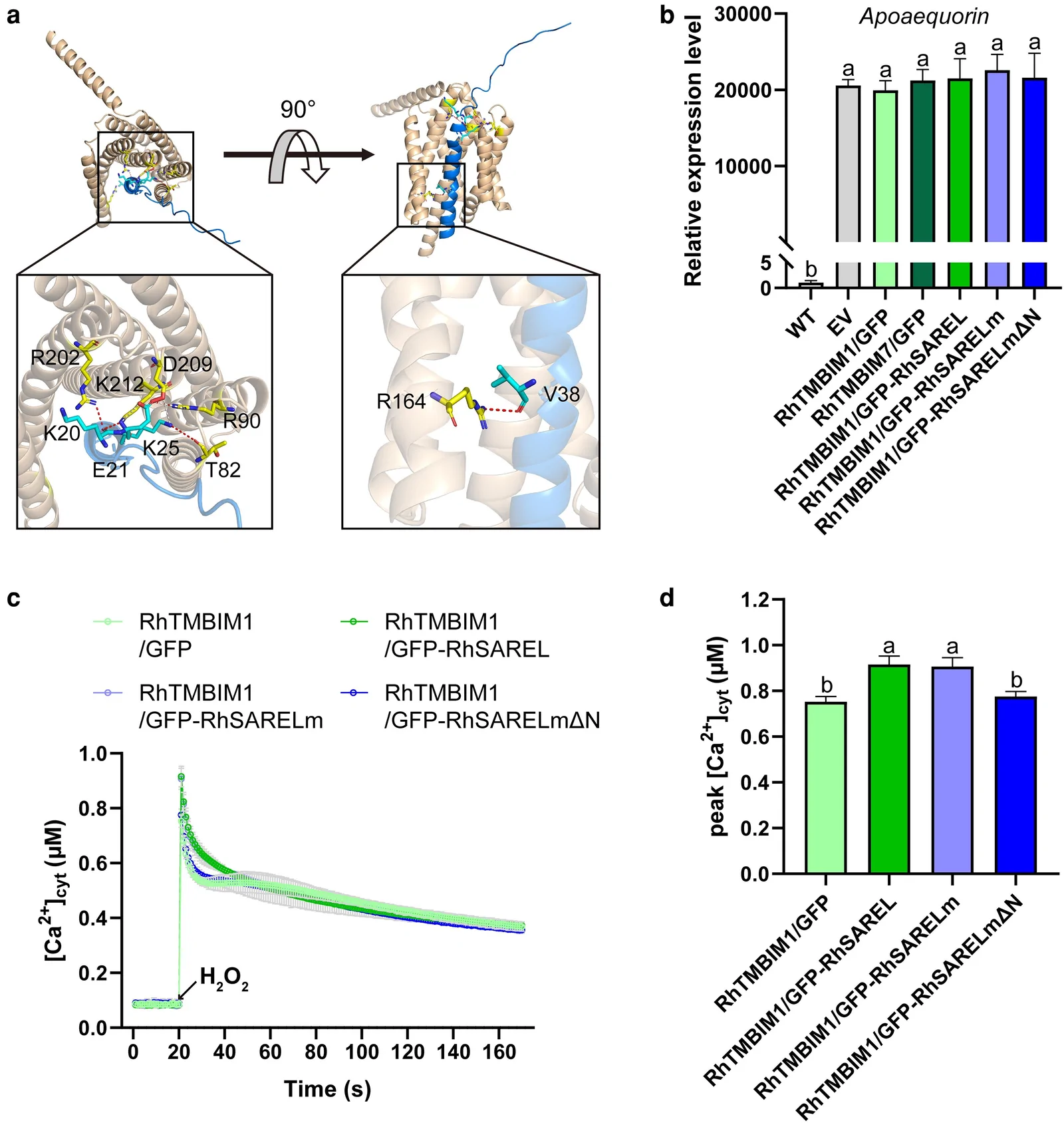
RhSAREL disrupts RhTMBIM1-mediated intracellular Ca^2+^ homeostasis. a) The interaction interface between the truncated variant RhSAREL_1-49_ and RhTMBIM1. The protein docking model of RhSAREL_1-49_ (marine blue) and RhTMBIM1 (wheat yellow) was predicted by GRAMM-X (https://gramm.compbio.ku.edu/), and the interaction interface was analyzed by PDBePISA (https://www.ebi.ac.uk/msd-srv/prot_int/). Interacting residues from RhTMBIM1 (yellow) and RhSAREL (cyan) are shown. Potential hydrogen bonds and salt bridges are depicted as red and white dotted lines, respectively. b) Expression of *apoaequorin* in different transgenic callus lines. Transgenic callus lines with comparable expression levels of *apoaequorin* were selected for further analysis. *RhUBI2* was used as a reference gene. Data are shown as means ± SD (n = 3). c) Time-course analysis of cytosolic Ca^2+^ dynamics in different transgenic callus lines after treatment with 5 mM H_2_O_2_. The luminescence signal was acquired at 1-s intervals. d) Quantification of the highest cytosolic Ca^2+^ concentration in c). Data are shown as means ± SD, n = 5 in c) and d). Different letters indicate statistically significant differences (*P* < 0.05) analyzed by one-way ANOVA with Tukey's test.

As RhTMBIM1 was not ubiquitinated by RhSAREL (Fig. S10a and b), we tried to explore the function of the interaction between RhTMBIM1 and RhSAREL. Based on the Y2H results (Fig. 3f and Fig. S7c) and the 3-dimensional (3D) structures of RhTMBIM1 and RhSAREL (Fig. S1b and S13a) predicted by ColabFold (Mirdita et al. 2022), we hypothesized that the transmembrane helix of RhSAREL is embedded in the barrel-like structure of RhTMBIM1. To test this hypothesis, protein docking between RhSAREL_1-49_ and RhTMBIM1 was performed using GRAMM-X (https://gramm.compbio.ku.edu/). The docking result revealed that RhSAREL_1-49_ resides within the barrel-like structure of RhTMBIM1. Interface analysis using PDBePISA (https://www.ebi.ac.uk/msd-srv/prot_int/) showed that 5 hydrogen bonds and 4 salt bridges are established by 4 amino acid residues of RhSAREL_1-49_ and six of RhTMBIM1 at the interaction interface between them (Fig. 5a). We found that 3 of the 4 residues from RhSAREL (Lys20, Glu21, Lys25) are located within its N-terminal 26-aa region. Moreover, we used 3 other web servers to perform the same protein docking analysis and obtained similar interacting conformations between RhSAREL_1-49_ and RhTMBIM1 (Fig. S13b–d).

Previous studies have demonstrated that BsYetJ, a TMBIM protein from *B. subtilis*, is a pH-sensitive Ca^2+^-leak channel and involved in maintaining Ca^2+^ homeostasis. The pore opening of BsYetJ channel and subsequent Ca^2+^ passage occur when Asp171 is transiently deprotonated and the cleft is electronegative under pH 7.4 conditions (Chang et al. 2014). The predicted 3D structure of RhTMBIM1 strikingly overlaps with that of BsYetJ, indicating a conserved channel structure in RhTMBIM1 (Fig. S13a). In the RhSAREL-RhTMBIM1 interaction complex, RhSAREL blocks the pore of RhTMBIM1 (Fig. 5a), which is presumed to prevent RhTMBIM1's Ca^2+^ leakage, thereby disrupting RhTMBIM1-mediated intracellular Ca^2+^ homeostasis. To test whether the interaction of RhSAREL and RhTMBIM1 is involved in regulating intracellular Ca^2+^ homeostasis, we individually generated transgenic rose calli overexpressing RhTMBIM1/GFP, RhTMBIM1/GFP-RhSAREL, RhTMBIM1/GFP-RhSARELm, or RhTMBIM1/GFP-RhSARELmΔN (Fig. 5b and Fig. S13e). We quantified [Ca^2+^]_cyt_ dynamics in calli using the Ca^2+^ reporter aequorin. It is known that H_2_O_2_ can induce an increase in [Ca^2+^]_cyt_ (Ihara-Ohori et al. 2007). After H_2_O_2_ treatment, an immediate Ca^2+^ spike was detected in all 4 callus lines (Fig. 5c). The RhTMBIM1/GFP-RhSAREL and RhTMBIM1/GFP-RhSARELm lines showed a higher amplitude of [Ca^2+^]_cyt_ increase compared to the RhTMBIM1/GFP control line. However, the calli co-expressing RhTMBIM1 and GFP-RhSARELmΔN (lacking the ability to interact with RhTMBIM1) exhibited a peak [Ca^2+^]_cyt_ comparable to that of the RhTMBIM1/GFP control line (Fig. 5c and d). These results indicate that RhSAREL disrupts the RhTMBIM1-mediated intracellular Ca^2+^ homeostasis by blocking its Ca^2+^-leak channel.

**Figure 6:**
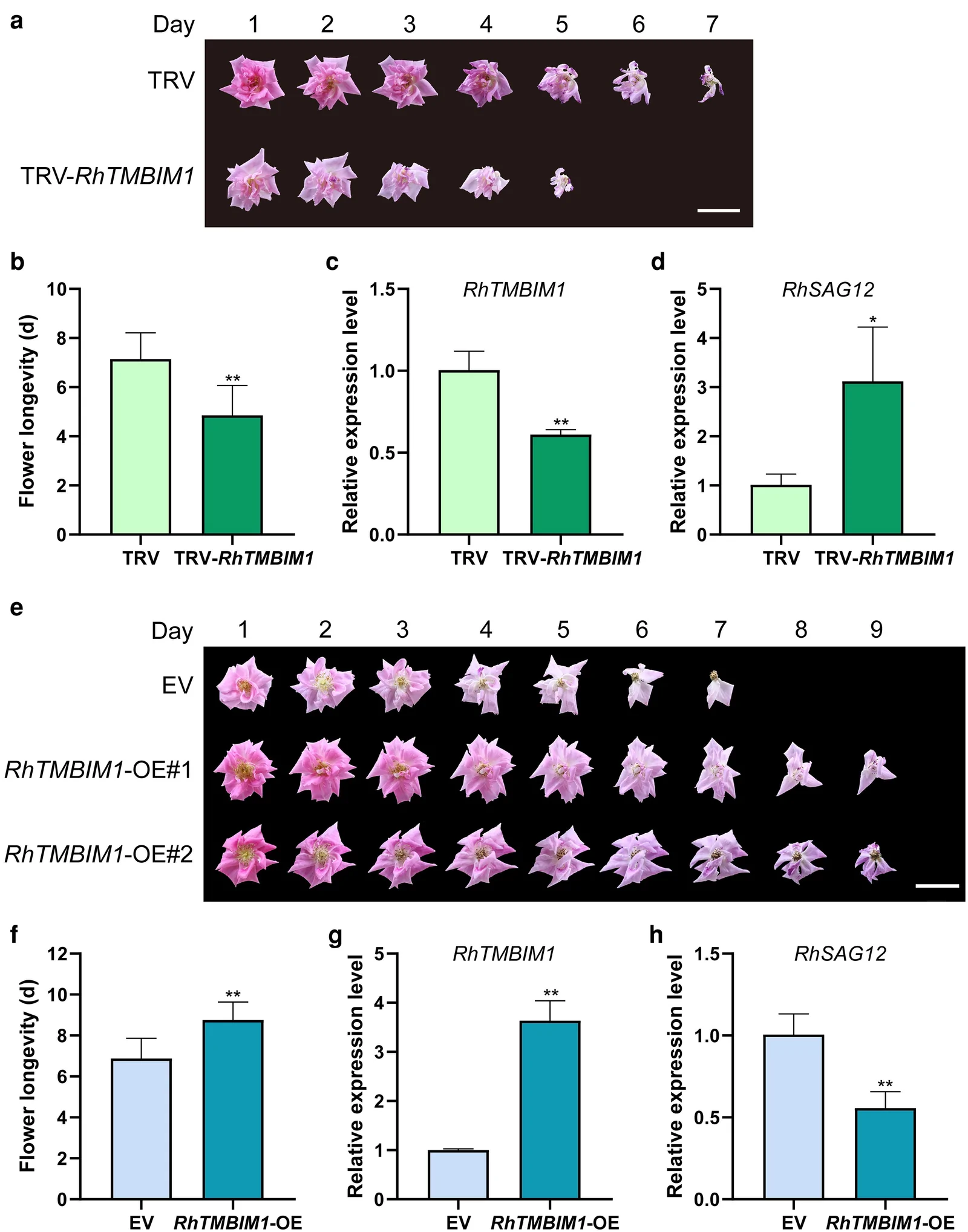
RhTMBIM1 delays petal senescence. a) Senescence phenotype of control and *RhTMBIM1*-silenced flowers. Bars, 5 cm. b) Flower longevity of control and *RhTMBIM1*-silenced flowers. c) Expression of *RhTMBIM1* in control and *RhTMBIM1*-silenced petals. d) Expression of senescence marker gene *RhSAG12* in control and *RhTMBIM1*-silenced petals. e) Senescence phenotype of control and *RhTMBIM1* overexpression flowers. Bars, 5 cm. f) Flower longevity of control and *RhTMBIM1* overexpression flowers. g) Expression of *RhTMBIM1* in control and *RhTMBIM1* overexpression petal. h) Expression of senescence marker gene *RhSAG12* in control and *RhTMBIM1* overexpression petals. *RhUBI2* was used as a reference gene. Data are shown as mean ± SD, n = 8 in b) and f), n = 3 in c), d), g), and h). Asterisks indicate statistically significant differences as determined by *t*-test (*P < 0.05, **P < 0.01).

To examine whether *RhTMBIM1* is involved in petal senescence, we silenced *RhTMBIM1* in rose flowers using VIGS (Fig. 6c). *RhTMBIM1*-silenced flowers exhibited more rapid senescence and increased expression of *RhSAG12* than the TRV control flowers (Fig. 6a, b, and d). In contrast, *RhTMBIM1*-overexpression led to delayed flower senescence and reduced *RhSAG12* transcript levels compared to the empty vector control (Fig. 6e–h). We also explored the function of *RhTMBIM1* in petal senescence-associated PCD using trypan blue staining and conductivity measurements. We found that silencing *RhTMBIM1* promoted petal senescence-associated PCD compared to the TRV controls, as evidenced by deeper trypan blue staining and significantly higher electrolyte leakage (Fig. S14a and c). On the contrary, overexpressing *RhTMBIM1* remarkably reduced petal PCD with slight staining and decreased electrolyte leakage (Fig. S14b and d). The expression of 3 PCD-associated genes (*RhBFN1*, *RhPASPA3*, and *RhEXI1*) was upregulated in *RhTMBIM1*-silenced petals and significantly repressed in *RhTMBIM1*-overexpressed petals (Fig. S14e and f). These results indicate that *RhTMBIM1* negatively regulates petal PCD and flower senescence.

**Figure 7:**
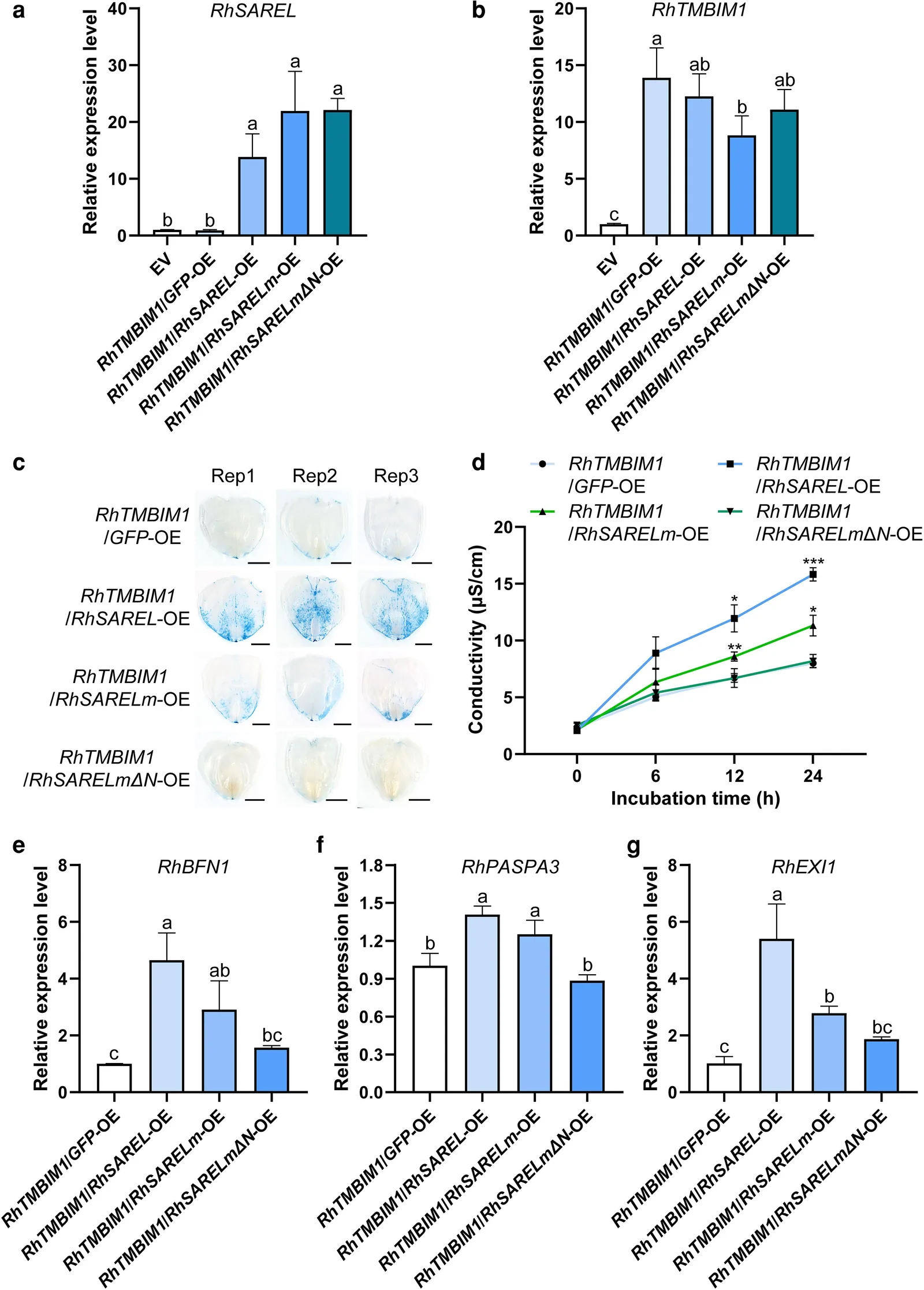
The interaction between RhSAREL and RhTMBIM1 accelerates petal senescence. a, b) Expression of *RhSAREL* (a) and *RhTMBIM7* (b) in petals under different co-expression combinations. RhTMBIM1-Flag was co-expressed with either GFP, GFP-RhSAREL, or GFP-tagged RhSAREL variants in rose petals. Co-expression of empty Flag-tagged and GFP-tagged vectors served as a negative control. *RhUBI2* was used as a reference gene. c) Trypan blue staining of petals under different co-expression combinations. d) Quantitative measurement of electrolyte leakages from petals under different co-expression combinations. e, f, g) Expression of PCD-associated genes in petals under different co-expression combinations. The outermost petals of infected flowers at stage 5 were sampled for phenotypic and gene expression analysis. Data are shown as mean ± SD (n = 3). For a) to b) and e) to g), different letters indicate statistically significant differences (*P* < 0.05) analyzed by one-way ANOVA with Tukey's test. For d), asterisks indicate statistically significant differences as determined by two-way ANOVA with Dunnett’s test (*P < 0.05, **P < 0.01, ***P < 0.001).

We further investigated whether the interaction of RhSAREL and RhTMBIM1 affects RhTMBIM1's role in regulating petal senescence. We transiently co-expressed *RhTMBIM1* with *RhSAREL* or its variants in rose petals (Fig. 7a and b) and analyzed the petal PCD phenotypes. Trypan blue staining revealed that co-expression of *RhTMBIM1* and *RhSAREL* led to more severe petal PCD than that of *RhTMBIM1* and *GFP*. When the variant RhSARELm lacking E3 ligase activity was co-expressed with RhTMBIM1, less petal PCD was observed in *RhTMBIM1*/*RhSARELm*-OE petals compared to *RhTMBIM1*/*RhSAREL*-OE ones. However, it was still more than that in *RhTMBIM1*/*GFP*-OE petals. The petals co-expressing RhTMBIM1 and the variant RhSARELmΔN (simultaneously lacking E3 ligase activity and the ability to interact with RhTMBIM1) showed similar PCD to that in *RhTMBIM1*/*GFP*-OE petals (Fig. 7c). Consistent with this, the highest electrolyte leakage and expressions of PCD-associated genes were detected in *RhTMBIM1*/*RhSAREL*-OE petals, while *RhTMBIM1*/*RhSARELmΔN*-OE petals exhibited comparable levels to *RhTMBIM1/GFP-*OE ones (Fig. 7d–g). Taken together, RhSAREL promotes rose flower senescence by disrupting RhTMBIM1-mediated Ca^2+^ homeostasis.

## Discussion

### RhSAREL is a positive regulator of rose flower senescence

Ubiquitination is widely involved in the regulation of plant growth and development, in which E3 ligase plays a central role based on its substrate recognition specificity (Callis 2014). E3 ligases are primarily classified into 4 types: HECT, RING, U­box, and cullin-RING ligases (CRLs) (Vierstra 2009). Different types of E3 ligases have been reported to participate in organ senescence regulation in plants. In Arabidopsis, the HECT-type E3 ligase *UPL5* delays leaf senescence through degradation of the transcription factor WRKY53 (Miao and Zentgraf 2010), while 2 U-box E3 ligases, *PUB12* and *PUB13*, promote dark-induced leaf senescence (Zhou et al. 2015). AtATL72, a RING-H2 E3 ligase, regulates the initiation of leaf senescence by monoubiquitinating the phosphatase SSPP, thereby inhibiting its dephosphorylation activity (Cao et al. 2025). Apple RING-HC E3 ligase MdSINA3 negatively regulates JA-mediated leaf senescence by ubiquitinating MdBBX37 for degradation (An et al. 2022). However, compared with their well-documented roles in leaf senescence, the functions of E3 ligases in flower senescence remain to be characterized. RING-HC protein MjXB3 was reported to accelerate flower senescence in *M. jalapa* (Xu et al. 2007). AtCOI1 promotes the degradation of AtJAZ3 and AtCO to trigger JA-induced flower senescence (Serrano-Bueno et al. 2022). RhSAF positively regulates ethylene-mediated flower senescence by ubiquitinating the GA receptor RhGID1s, leading to their subsequent degradation (Lu et al. 2024). Both AtCOI1 and RhSAF are F-box proteins, subunits of CRLs.

Here, we identified RhSAREL as a RING-H2 E3 ligase (Fig. 1c and Fig. S1b), which is the largest subtype of RING-type E3 ligases (Stone et al. 2005). The expression of *RhSAREL* was ethylene-dependent, as it was significantly induced by ethylene treatment but suppressed by 1-MCP (Fig. 1b). *RhSAREL* acts downstream of ethylene signaling via a floral senescence regulatory pathway distinct from that of *RhSAF*. In addition, *RhSAREL* is highly expressed in early floral senescence (Fig. 1a), while *RhSAF* is in the late stage, suggesting a potential role of *RhSAREL* in floral senescence initiation. Consistent with this, single-nucleus transcriptomic leaf data revealed that the transcript level of *AtATL66* (an ortholog of *RhSAREL* from Arabidopsis) peaked at stage 3 in epidermal cells, when the leaves still appear green, but leaf senescence has already been initiated (Guo et al. 2025). These results suggest that *RhSAREL*, a RING-H2 E3 ligase, responds to ethylene signals and may function in the initiation of flower senescence.

### RhSAREL promotes flower senescence via both E3 ligase-dependent and -independent pathways

Senescence is a strictly regulated developmental process. A multilayered regulatory network ensures orderly and timely onset and progression of senescence. Previous studies have demonstrated that *BI-1* is induced during flower senescence in *Brassica oleracea* and *Ipomoea nil* (Coupe et al. 2004; Yamada et al. 2009). These data suggest that *BI-1* may play a role in flower senescence. We found that there are a total of 8 homologs of *BI-1* in the rose genome (Fig. S6). Transcriptome data and RT-qPCR analysis showed that RhTMBIM1 and RhTMBIM7 are the predominant TMBIM family genes during flower senescence and maintain stable expression levels, a pattern that differs from previous reports on *BI-1* genes in other species (Table S2, Fig. S8). Silencing *RhTMBIM1* or *RhTMBIM7* resulted in accelerated flower senescence, indicating that both of them are senescence inhibitors (Fig. 6a–d and Fig. S11). Given that the E3 ligase RhSAREL interacts with RhTMBIM1 and RhTMBIM7, they are possibly regulated in a post-translational manner. Our results further revealed that ethylene-induced RhSAREL promotes flower senescence by regulating RhTMBIM1 and RhTMBIM7 via E3 ligase-independent and -dependent pathways. On the one hand, RhSAREL ubiquitinates RhTMBIM7 and promotes its degradation through the 26S proteasome pathway (Fig. 4a–e). On the other hand, RhSAREL interacts with RhTMBIM1 via its N-terminal 26-aa region but does not affect the level of RhTMBIM1's ubiquitination (Fig. 3a–c, Fig. S10a and b). RhSAREL disrupts RhTMBIM1-mediated Ca^2+^ homeostasis by blocking RhTMBIM1's pore responsible for Ca^2+^ leakage (Figs. 5 and 7). Generally, the *SAG12* gene is regarded as a molecular marker of organ senescence (Gan and Amasino 1997). The expression of *RhSAG12* in TRV-*RhTMBIM7* petals was significantly upregulated, indicating the role of RhTMBIM7 in delaying flower senescence (Fig. S11d). When petal senescence is initiated, ethylene-induced RhSAREL promotes the ubiquitination and degradation of RhTMBIM7 to ensure timely senescence. We observed restored *RhSAG12* expression in the TRV-*RhSAREL*/*RhTMBIM7* petals close to the empty vector control (Fig. S12e), indicating that the RhSAREL-RhTMBIM7 module is involved in the precise temporal regulation of rose petal senescence.

Interestingly, protein sequence alignment revealed that the residues of RhSAREL involved in interacting with RhTMBIM1 belong to a novel motif (RGR), located in the N-terminal 26-aa region of RhSAREL, and are conserved in multiple species (Figs. 1c and 5a). Furthermore, this motif is part of sequence LOGO 80, which was generated from the alignment of ATL proteins across 24 plant species (Aguilar-Hernández et al. 2011). Despite these results above, it still requires further investigation whether these conserved RGR motifs would function to interact with their counterpart TMBIMs in other plant species. RhSAREL ubiquitinates RhTMBIM7, it does not ubiquitinate another TMBIM protein, RhTMBIM1. This may be attributed to the low sequence identity (19.5%) between RhTMBIM1 and RhTMBIM7 (Fig. S15). There are also some different distribution patterns of lysine residues responsible for ubiquitination between TMBIM1 and TMBIM7 homologous proteins (Fig. S18).

### RhTMBIM1 and RhTMBIM7 prolong flower longevity by maintaining intracellular Ca^2+^ homeostasis

TMBIM family proteins have been reported to regulate plant growth and mediate responses to biotic and abiotic stresses. For example, AtLFG2 promotes cell elongation by suppressing the degradation of the BR receptor brassinosteroid insensitive 1 (BRI1) (Yamagami et al. 2017). *AtBI-1* overexpression improves drought tolerance in sugarcane, as *AtLFG1*/*2*/*3*/*5* do in Arabidopsis (Ramiro et al. 2016; Zhou et al. 2025). AtLFG1/2 and PvBI-1a/GmBI-1α increase plant susceptibility to powdery mildew and rhizobia, respectively (Weis et al. 2013; Hernández-López et al. 2019; Yuan et al. 2023). Additionally, AtBI-1 reduces disease tolerance to *Pseudomonas syringae*, whereas *AtBI-1* mutants show improved systemic movement of potexviruses and potyviruses (Gaguancela et al. 2016; Adhikari et al. 2024; Tang et al. 2024b). Here, we demonstrated that RhTMBIM1 and RhTMBIM7 prolong rose flower longevity (Fig. 6 and Fig. S11). Silencing *RhTMBIM1* led to significant PCD in petals, while overexpression of *RhTMBIM1* suppressed it (Fig. S14).

TMBIM family proteins from animals, plants, and bacteria were reported to maintain intracellular Ca^2+^ homeostasis (Ihara-Ohori et al. 2007; Rojas-Rivera et al. 2012; Chang et al. 2014). In humans, all TMBIM proteins possess the activity of mediating Ca^2+^ leakage from the ER, which contributes to intracellular Ca^2+^ homeostasis (Lisak et al. 2015). Two aspartate residues critical for TMBIM protein function are strictly conserved in TMBIM homologs from rose (*Rosa hybrida*) and human (*Homo sapiens*) (Chang et al. 2014) (Fig. S15). RhTMBIM1 shares a similar channel structure with BsYetJ, which has been characterized as a Ca^2+^ leakage channel (Fig. S13a). Therefore, RhTMBIM1 may also act as a Ca^2+^ leakage channel and regulate intracellular Ca^2+^ homeostasis. In our study, co-expressing RhTMBIM1 with RhSAREL or RhSARELm significantly increased the [Ca^2+^]_cyt_ levels in rose callus cells compared to the control following H_2_O_2_ treatment (Fig. 5b–d). This was attributable to the interaction between RhTMBIM1 and RhSAREL, which disrupted the RhTMBIM1-mediated Ca^2+^ homeostasis. Consistent with this, increased PCD was detected in petals co-expressing *RhTMBIM1* with *RhSAREL* or *RhSARELm* (Fig. 7), indicating that Ca^2+^ homeostasis is critical to rose flower longevity. A sharp elevation of [Ca^2+^]_cyt_ levels was also observed in senescent root cap cells, indicating the disruption of intracellular Ca^2+^ homeostasis (Wang et al. 2024). It has been proved that the elevated [Ca^2+^]_cyt_ activated downstream signaling pathways to accelerate organ senescence in plants (Edel et al. 2017; Durian et al. 2020; Chen et al. 2023). Arabidopsis TMBIM protein AtBI-1 delays MeJA-induced leaf senescence by regulating intracellular Ca^2+^ homeostasis (Yue et al. 2012). We also compared the 3D structure of RhTMBIM7 with RhTMBIM1 or BsYetJ (Fig. S16). RhTMBIM7 shared a channel structure with these 2 TMBIM proteins, indicating that RhTMBIM7 may mediate Ca^2+^ homeostasis as they do. We generated transgenic rose calli overexpressing EV or RhTMBIM7/GFP (Fig. 5b and Fig. S13e). We found that both RhTMBIM1/GFP and RhTMBIM7/GFP transgenic calli exhibited a diminished increase in [Ca^2+^]_cyt_ after H_2_O_2_ treatment compared with EV calli (Fig. S17). Collectively, these data suggest that RhTMBIM1 and RhTMBIM7 could prolong rose flower longevity by maintaining intracellular Ca^2+^ homeostasis, which is functionally antagonized by RhSAREL.

**Figure 8:**
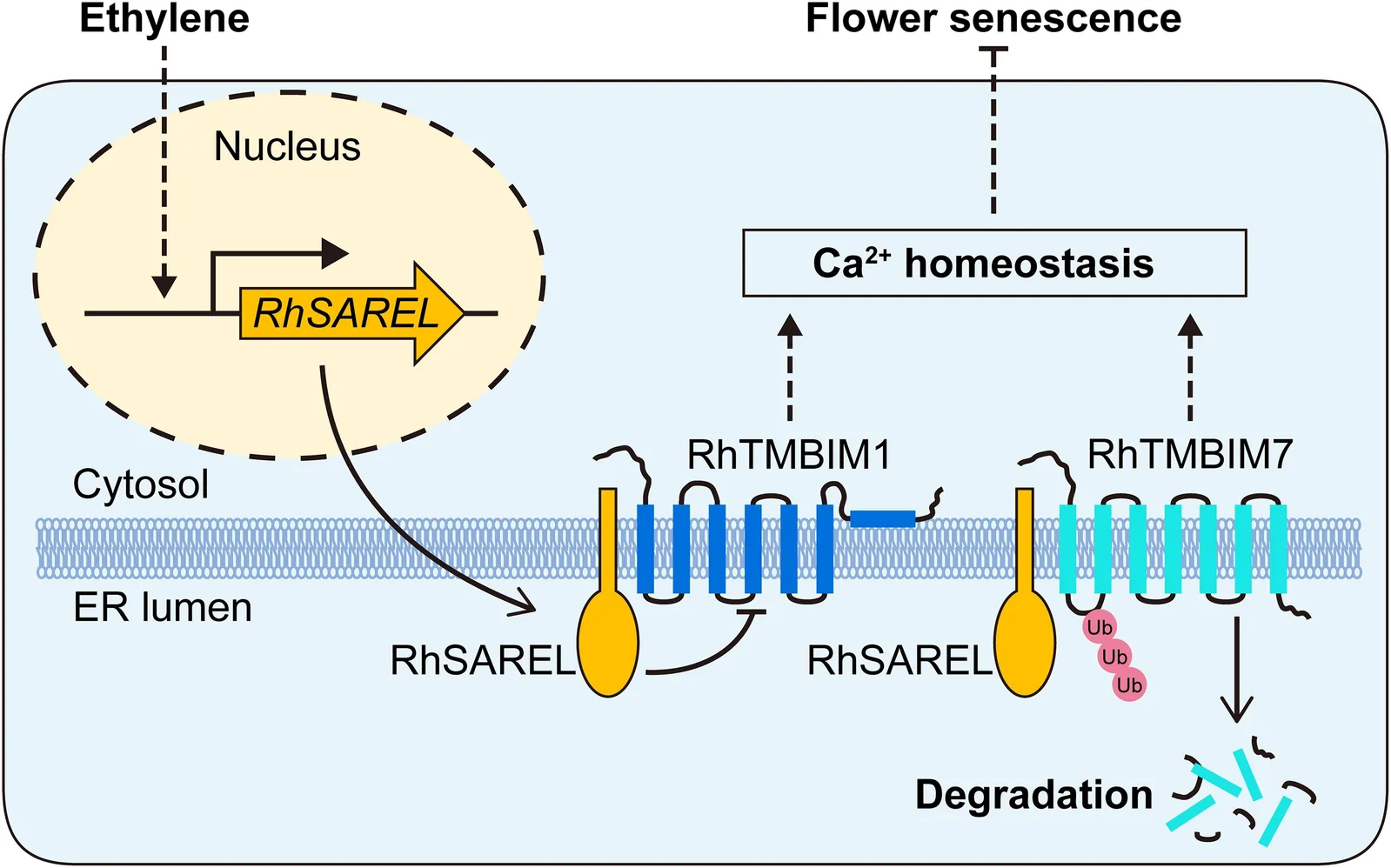
A proposed model of RhSAREL's role in rose flower senescence. At the early stage of rose flower senescence, the transcript level of *RhSAREL* is induced by increased ethylene. RhSAREL ubiquitinates RhTMBIM7 and promotes its degradation via the 26S proteasome pathway. In addition, RhSAREL disrupts RhTMBIM1-mediated Ca^2+^ homeostasis by predictably occluding its Ca^2+^ leakage channel. Both RhTMBIM1 and RhTMBIM7 function to delay flower senescence through maintaining Ca^2+^ homeostasis, which is antagonized by RhSAREL's roles.

Based on these results, we propose that the constitutively expressed *RhTMBIM1* and *RhTMBIM7* maintain intracellular Ca^2+^ homeostasis in rose petals before flower senescence. Increased ethylene levels activate the expression of *RhSAREL*. RhSAREL promotes flower senescence by interacting and regulating RhTMBIM1 and RhTMBIM7 via 2 distinct pathways: first, it ubiquitinates RhTMBIM7 for degradation, and second, it disrupts Ca^2+^ homeostasis by blocking the pore of RhTMBIM1 (Fig. 8).

## Materials and methods

### Plant materials and growth conditions

The rose used in this study was a rose (*R. hybrida*) cultivar “Samantha”. Arabidopsis (*A. thaliana*) ecotype Col-0 and tobacco (*N. benthamiana*) were also used. All plantlets were grown in soil under a long-day condition (16-h light/8-h dark) at ∼22°C and ∼70% relative humidity. Flower opening stages were defined previously (Wu et al. 2017).

### RNA extraction and RT-qPCR analysis

The total RNA of rose petals was extracted using the hot borate method as previously described (Fan et al. 2020). The cDNA was synthesized using HiScript II Q RT SuperMix (Vazyme, China). The reverse transcription-quantitative PCR (RT-qPCR) was conducted using Realtime PCR Super mix (Mei5 Biotechnology, China) on a StepOnePlus Real-Time PCR system (Applied Biosystems, USA). *RhUBI2* (XM_024320418) was used as a reference gene.

### Subcellular localization

The CDS of *RhSAREL* was inserted into the pSuper1300-GFP vector to generate the *pSuper::GFP-RhSAREL* expression construct. The ER-rk *CD3-959* vector was used as the endoplasmic reticulum (ER) marker (Nelson et al. 2007). *A. tumefaciens* (GV3101) carrying *pSuper::GFP-RhSAREL* or *pSuper::GFP* and a marker vector were co-infiltrated into tobacco leaves. Fluorescence signals were imaged 72 h after infiltration using a confocal laser-scanning microscope (Zeiss, Germany). GFP was excited at 488 nm and detected at 505 to 550 nm, and mCherry was excited at 552 nm and detected at 575 to 630 nm.

### Protein expression and purification

RING domain mutation of RhSAREL (RhSARELm, C123A/H125A/H128A) was generated as previously reported (Stone et al. 2005). RhSAREL and RhSARELm were introduced into the pMAL-cR1 vector to produce recombinant proteins with the maltose-binding protein (MBP)-tag. AtUBA2 (E1)-6×His and AtUBC10 (E2)-6×His in pET-28a vectors were kindly provided by Feifei Yu (China Agricultural University). Protein expression was conducted in *Escherichia coli* strain BL21. Transformed cells were cultured at 37°C to an OD600 of 0.5 to 0.8 and induced by 0.2 mM isopropyl β-D-thiogalactoside (IPTG) at 16°C for 16 h. The MBP-tagged proteins were purified by Amylose Resin (NEB, USA). The histidine (His) tagged-proteins were purified by Ni Sepharose 6 Fast Flow (Cytiva, USA).

### *In vitro* ubiquitination assay

The in vitro ubiquitination assay was performed as previously reported (Yu et al. 2020). 50 ng purified AtUBA2 (E1)-6 × His, 200 ng purified AtUBC10 (E2)-6 × His, 5 µg recombinant human ubiquitin (UB-biotech, China), and 500 ng purified MBP-RhSAREL or MBP-RhSARELm were added to the reaction buffer (50 mM Tris-HCl pH 7.4, 2 mM ATP, 10 mM MgCl_2_, 2 mM DTT). For in vitro ubiquitination assays of 2 TMBIM proteins, 3 × Flag-tagged TMBIM proteins were immunoprecipitated using anti-Flag magnetic beads (LABLEAD, China) from total protein extract of tobacco leaves and divided equally into different reactions. All reactions were conducted at 30°C with gentle agitation for 90 min. Anti-Ub or anti-Flag antibodies (ABclonal, China) were used to detect the poly-ubiquitination of MBP-RhSAREL, RhTMBIM1-Flag, and RhTMBIM7-Flag.

### VIGS

The VIGS assay was conducted as previously reported with minor modifications (Jiang et al. 2023). Gene-specific fragment of *RhSAREL* (400 bp), *RhTMBIM1* (400 bp), or *RhTMBIM7* (400 bp) was constructed in the pTRV2 vector. pTRV1, pTRV2, and these constructs were transformed into Agrobacterium (*A. tumefaciens*) strain GV3101. The transformed Agrobacterium cells were cultured at 28°C for 14 h and resuspended in the infiltration buffer (10 mM MES, 10 mM MgCl_2_, and 200 µM acetosyringone, pH 5.6) after centrifuging. The resuspended cell cultures were kept in the dark at room temperature for at least 3 h. The culture of TRV2 or each recombinant TRV2 was mixed with TRV1 in a 1:1 (v/v) ratio. One-year-old rose plantlets grown in soil were used for Agrobacterium infection with visible flower buds. The rose flower buds and leaves were immersed in the Agrobacterium mixture under a vacuum of −98 kPa for 10 min. After infiltration, all plantlets were grown under normal condition. Flower senescence phenotypes were recorded approximately 2 wk after infiltration.

### Transient overexpression in rose

The transient overexpression in rose was performed as described previously (Chen et al. 2023). The full length CDS of *RhSAREL* or *RhTMBIM1* was inserted to the pSuper1300 vector. The Agrobacterium strain GV3101 harboring the construct of *pSuper::RhSAREL* or *pSuper::RhTMBIM1* was resuspended to a OD600 of 1.0 with the infiltration buffer mentioned above. The rose flowers at stage 1 were immersed in the Agrobacterium solution under a vacuum of −98 kPa for 10 min. Infiltrated plantlets were grown under the normal condition. Rose flowers were used for functional analysis at 3 d post-infiltration.

For co-expression of RhTMBIM7-Flag and GFP in rose petals, the resuspended Agrobacterium solution of RhTMBIM7-Flag was mixed with GFP in a 2:1 (v/v) ratio. The outer whorl petals at Stage 2 were injected with Agrobacterium mixture using a 1-mL medical syringe needle. Two days after infection, flowers were treated with air or 10 μL/L ethylene for 24 h and then collected. The abundance of RhTMBIM7-Flag protein was assessed by immunoblotting using an anti-Flag antibody. GFP was used as an internal control. Transcript levels of *RhTMBIM7* and *RhSAREL* were analyzed by RT-PCR with *RhUBI2* as the internal reference. The PCR amplification was performed for 30 cycles with the same primers as RT-qPCR (Table S3), and *RhTMBIM7* was amplified for 24 cycles.

### Conductivity measurement

Infected petals were harvested at stage 5. Two discs of 1 cm diameter were cut from 1 petal using a hole puncher. These 2 discs were put into a 5 mL sterilized centrifuge tube and incubated with 2 mL sterilized ultrapure water for 5 min. The water in the tube was replaced with 2 mL of fresh sterilized ultrapure water. Petal discs were incubated in the light at ∼22°C. Conductivity of the solution was measured by a conductivity meter (YuePing, China) at the determined time points.

### Trypan blue staining

The trypan blue staining was performed as previously described (Fernández-Bautista et al. 2016). The outermost petals were collected from inoculated flowers at stage 5 and placed in culture plates. Petals were incubated with 5 mL of fresh trypan blue solution (10 mg/mL trypan blue solid, 25% lactic acid, 25% phenol, 25% glycerol) for 30 min at room temperature. The trypan blue solution was replaced with 5 mL of 100% ethanol. Petals were incubated with the ethanol solution until red tissue had become completely colorless. Petals stained with trypan blue were placed in 5 mL centrifuge tubes containing 60% glycerol solution for subsequent observation.

### Rose transformation

For generating *RhSAREL*-RNAi transgenic rose lines, a 300 bp gene-specific fragment of *RhSAREL* was inserted into the pTCK303 vector. The construct was introduced into Agrobacterium strain EHA105. Somatic embryos of rose were used for *A. tumefaciens*-mediated transformation (Liu et al. 2021). Sterile rooted cuttings of EV and *RhSAREL*-RNAi transgenic lines were transplanted into soils. The flower phenotypes were recorded around 2 mo after transplanting. For generating transgenic rose calli, fusion sequences of 2A-apoaequorin-2A-GFP, RhTMBIM1-2A-apoaequorin-2A-GFP, RhTMBIM7-2A-apoaequorin-2A-GFP, RhTMBIM1-2A-apoaequorin-2A-GFP-RhSAREL, RhTMBIM1-2A-apoaequorin-2A-GFP-RhSARELm, and RhTMBIM1-2A-apoaequorin-2A-GFP-RhSARELΔN were synthesized using multiple rounds of overlapping PCR. These sequences were cloned into the pSuper1300 vector and subsequently introduced into the Agrobacterium strain EHA105. Rose calli maintained in the laboratory were used for *A. tumefaciens*-mediated transformation (Cheng et al. 2021). The *apoaequorin* gene was kindly provided by Yang Zhao. Transgenic calli were used for [Ca^2+^]_cyt_ measurements after 2 mo of selective proliferation.

### Yeast two-hybrid assay

The yeast two-hybrid assay was performed using the DUALmembrane system (Dualsystems Biotech, Switzerland). The bait proteins, including RhSAREL and its truncated variants, were cloned into the pBT3-STE vector. The prey proteins, including RhTMBIM1, RhTMBIM7, and truncated variants of RhTMBIM1, were cloned into the pPR3-N vector. Pairs of vectors were co-transformed into yeast strain NMY51. The combination of pBT3-STE-RhSAREL and pOst1-Nub1 was used as a positive control. Combinations of each constructed pBT3-STE vector with the empty pPR3-N vector were used as negative controls. Positive transformants were adjusted to OD600 = 0.1 and then subjected to serial 10-fold dilutions (undiluted, 10^−1^, 10^−2^). The yeast cell suspension was spotted onto SD-Trp-Leu plates and SD-Trp-Leu-His-Ade plates containing 40 μg/mL X-Gal (5-Bromo-4-chloro-3-indolyl β-D-galactopyranoside) and 10 mM 3-AT (3-amino-1,2,4-triazole), and the plates were incubated at 30°C for 3 to 5 d.

### Co-immunoprecipitation assay

GFP-tagged proteins were co-expressed with Flag-tagged proteins in tobacco leaves for 3 d. Co-expression of each Flag-tagged protein and GFP served as a negative control. The infected leaves were treated with 50 μM MG132 12 h before harvest. Total protein was extracted using cell lysis buffer containing 50 μM MG132. After 40 μL of the lysate was removed for input controls, the total protein extract was immunoprecipitated with GFP-Trap magnetic beads (ChromoTek, Germany) at 4°C for 4 h. The beads were washed 3 times and then incubated in SDS loading buffer at 95°C for 10 min. The immunoblotting analysis was carried out with anti-GFP (Beyotime, China) or anti-Flag antibody.

### Split-luciferase complementation assay

The CDS region of *RhSAREL* was constructed into the pCAMBIA1300-cLUC vector. The CDS regions of *RhTMBIM1* or *RhTMBIM7* were inserted into the pCAMBIA1300-nLUC vector. Those vectors were transformed into Agrobacterium strain GV3101. Defined combinations of constructs were expressed in tobacco leaves for 3 d. The infiltrated leaves were detached and then treated with 1 mM luciferin in the dark for 10 min. The images were captured by a chemiluminescence imaging system (Tanon, China).

### *In vivo* ubiquitination assay

GFP-RhSAREL was co-expressed with RhTMBIM1-Flag or RhTMBIM7-Flag in tobacco leaves. Expression of RhTMBIM1-Flag or RhTMBIM7-Flag alone, or co-expression of each with GFP, served as negative controls. 50 μM MG132 was injected into the infiltrated leaves 2.5 d after infiltration. Leaves were harvested 3 d after infiltration. Total protein was extracted using cell lysis buffer (Beyotime, China). The protein levels of RhTMBIM7-Flag, GFP, and GFP-RhSAREL in infiltrated tobacco leaves were assessed by western blotting prior to immunoprecipitation. Samples with sufficiently expressed proteins were used for immunoprecipitation with anti-Flag magnetic beads. The ubiquitination of RhTMBIM1 and RhTMBIM7 was detected by anti-Ub (kindly provided by Feifei Yu) or anti-Flag antibody.

### Semi-in vivo protein degradation assay

The semi-in vivo protein degradation assay was performed as previously described (Liu et al. 2010). RhTMBIM1-Flag, RhTMBIM7-Flag, GFP-RhSAREL, and GFP were expressed in tobacco leaves. Three days after infiltration, all samples were harvested and separately extracted in native extraction buffer (50 mM Tris-MES pH 8.0, 0.5 M sucrose, 1 mM MgCl_2_, 10 mM EDTA, 5 mM DTT, protease inhibitor cocktail). Total protein extraction of GFP-RhSAREL was mixed with that of RhTMBIM1-Flag or RhTMBIM7-Flag. The combinations of GFP and RhTMBIMs served as negative controls. 10 μM ATP was added to the reaction. The mixtures were incubated at 4°C with gentle agitation for 8 h. Equal volumes of samples were collected at the determined time points. The immunoblotting analysis was conducted using anti-Actin (Bio Basic Inc., Canada) or anti-Flag antibody. For assays with MG132 treatment, RhTMBIM7-Flag protein samples from new biological replicates were used, and a final concentration of 50 μM MG132 was added.

### [Ca^2+^]_cyt_ measurement

[Ca^2+^]_cyt_ measurements were performed according to a previously reported method (Sun et al. 2021a). The transformed calli, which had been sub-cultured for 3 d, were placed in 1.5 mL sterilized microcentrifuge tubes and immersed in 200 μL of 10 µM coelenterazine (MedChemExpress, USA) solution. The calli were then incubated in the dark for 12 h. The background luminescence signal was recorded for 60 s at 1-s intervals using a luminometer (Promega, USA). Next, 200 μL of 10 mM H_2_O_2_ solution was added into the tube, and the luminescence signal was immediately recorded for 150 s. Finally, 400 μL of the discharge buffer (2 M CaCl_2_ in 20% ethanol) was added into the tube, and the luminescence signal was recorded for 120 s. [Ca^2+^]_cyt_ was calculated as previously described (Knight et al. 1996).

### Statistical analysis

Statistical significance was determined using Student's *t*-test, one-way ANOVA, or two-way ANOVA with GraphPad Prism 8.0 software (GraphPad Software Inc., San Diego, CA, USA).

### Accession numbers

Gene sequences from this study can be found in GenBank (http://www.ncbi.nlm.nih.gov/genbank) under the following accession numbers: *RhSAREL* (XM_024308358), *RhTMBIM1* (XM_024314737), *RhTMBIM7* (XM_024341039), *RhSAG12* (XM_024301607), *RhUBI2* (XM_024320418), *RhBFN1* (XM_024304253), *RhPASPA3* (XM_024332342), *RhEXI1* (XM_040511638).

## Supplementary Material

kiag587_Supplementary_Data

## Data Availability

All data generated during this study are included in this article and the Supplemental data.
