# Supplementary material for: RING-type E3 ligase RhSAREL promotes flower senescence by differentially regulating two TMBIM family members, RhTMBIM1 and RhTMBIM7, in Rosa hybrida
Source: Plant Physiol. 2026 Aug 11;201(4):kiag587. doi: 10.1093/plphys/kiag587 (PMC13521286; doi:10.1093/plphys/kiag587)
Supplement: Supplementary file 1 [file kiag587_supplementary_data.zip › Supplemental Figures-Rose-RhSAREL-Flower senescence.docx]

**Figure S1. *RhSAREL* is homologous to *AtATL66*.**

A) Phylogenetic analysis of ATL homologs in *Rosa hybrida* and *Arabidopsis thaliana*. The phylogenetic tree was constructed using the neighbor-joining method in MEGA X with 1000 bootstrap replicates. The scale bar indicates the branch length. RchiOBHm_Chr1g0370601 (RhSAREL) is marked with a black arrowhead.

B) Predicted 3D structure of RhSAREL. The prediction of protein structure was conducted using ColabFold (https://github.com/sokrypton/ColabFold). The panel on the right is the same as the panel on the left, but rotated 90° counterclockwise in the horizontal plane. The transmembrane region and the RING-H2 domain of RhSAREL are shown in blue and red, respectively.

**Figure S2. Expression profile of RING-H2 type E3 ligases differentially expressed during petal senescence.**

A) Phylogenetic analysis of RING-H2 type E3 ligases in rose. The phylogenetic tree was constructed using the neighbor-joining method in MEGA X with 1000 bootstrap replicates. The scale bar indicates the branch length.

B) Heatmap of upregulated and downregulated RING-H2 type E3 ligases identified by RNA-seq at different flower opening stages. RING-H2-type E3 ligases with a fold change (Stage 5 vs. Stage 2) > 1.5 or < 0.67 (FDR < 0.05) and a mean FPKM (across stages 2 to 5) > 10 were selected for heatmap generation. The relative expression levels [log_2_(FPKM)] are shown in the heatmap, with the color scale representing the expression values. The gene *RchiOBHm_Chr1g0370601* (*RhSAREL*) is highlighted in red.

**Figure S3. E3 ligase activity of RhSAREL *in vitro*.**

The self-ubiquitination of RhSAREL and its mutant was detected in the presence of AtUBA2 (E1)-6×His, AtUBC10 (E2)-6×His, and Ub. MBP served as a negative control.

**Figure S4. Silencing *RhSAREL* delays petal senescence.**

A) Senescence process of control and *RhSAREL*-silenced rose flowers. Bars, 5 cm.

B) Flower longevity of control and *RhSAREL*-silenced rose flowers. Data are shown as means ± SD (n=10).

C) Expression of *RhSAREL* in control and *RhSAREL*-silenced petals.

D) Expression of senescence marker gene *RhSAG12* in control and *RhSAREL*-silenced petals.

*RhUBI2* was used as a reference gene. Data are shown as means ± SD, n=5 in C) and D). Asterisks indicate statistically significant differences as determined by *t*-test (**P<0.01, ****P<0.0001).

**Figure S5. *RhSAREL* promotes cell death during petal senescence.**

A, B) Trypan blue staining of rose petals with transient silencing (A) or overexpression (B) of *RhSAREL*. Bars, 1 cm.

C, D) Quantitative measurement of ion leakages from rose petals with transient silencing (C) or overexpression (D) of *RhSAREL*.

E, F) Expression of PCD-associated genes in petals with transient silencing (E) or overexpression (F) of *RhSAREL*.

The outermost petals of infected flowers at stage 5 were sampled for phenotypic and gene expression analysis. *RhUBI2* was used as a reference gene. Data are shown as means ± SD, n=3 in C)–F). Asterisks indicate statistically significant differences as determined by two-way ANOVA with Sidak’s test (*P<0.05, **P<0.01, ***P<0.001, ****P<0.0001).

**Figure S6. Identification of the TMBIM protein family in rose**.

Phylogenetic analysis of rose TMBIM proteins and their homologs in other species. The phylogenetic tree was constructed using the neighbor-joining method in MEGA X with 1000 bootstrap replicates. The scale bar indicates the branch length. Members of rose TMBIM family are highlighted in red. Rh, *Rosa hybrida*; At, *Arabidopsis thaliana*; Hs, *Homo sapiens*.

**Figure S7. Analysis of the Interaction between RhSAREL and RhTMBIM1.**

A) Interaction of RhSARELΔN with RhTMBIM1 in Co-IP assays. RhSARELΔN is a truncated variant of RhSAREL lacking the N-terminal 26-aa region.

B) Interaction of RhSARELΔN with RhTMBIM1 in Co-IP assays.

C) Interaction of RhTMBIM1 or its truncated variants with RhSAREL in Y2H assays. The domain of RhTMBIM1 was analyzed by SAMART (https://smart.embl.de/). Transformed yeast cells were serially diluted and cultured on SD-Trp-Leu medium as well as SD-Trp-Leu-His-Ade medium supplemented with 40 μg/mL X-Gal and 10 mM 3-AT.

**Figure S8. RT-qPCR analyses of *RhTMBIM1* and *RhTMBIM7* expression in petals at different flower opening stages.**

*RhUBI2* was used as a reference gene. Data are shown as means ± SD (n=3). Different letters indicate statistically significant differences (P＜0.05) analyzed by one-way ANOVA with Tukey’s test.

**Figure S9. The native anti-MBP blot in Figure 4A.**

Samples from different reaction combinations of the *in vitro* ubiquitination assay were immunoblotted with anti-MBP antibody.

**Figure S10. RhSAREL neither ubiquitinates RhTMBIM1 nor affects its degradation.**

A) *In vitro* ubiquitination assay of RhTMBIM1. MBP and MBP-RhSARELm served as negative controls. The immunoprecipitated RhTMBIM1-Flag from tobacco leaves was used as the substrate. Immunoblot analysis was performed using anti-Flag, anti-Ub, and anti-MBP antibodies. RhTMBIM1-Flag-Ubn denotes the ubiquitinated RhTMBIM1-Flag protein bands.

B) *In vivo* ubiquitination assay of RhTMBIM1. *RhTMBIM1-Flag* was transiently co-expressed with *GFP* or *GFP-RhSAREL* in tobacco leaves for 3 days. The leaves were treated with 50 μM MG132 12 hours before harvest. Total protein was extracted and immunoprecipitated using anti-Flag magnetic beads. The immunoblotting analysis was conducted with anti-Flag and anti-Ub antibodies.

C) Semi-*in vivo* protein degradation assay of RhTMBIM1. The protein extract from tobacco leaves expressing *RhTMBIM1-Flag* was mixed with the protein extract from leaves expressing *GFP* or *GFP-RhSAREL* and incubated at 4 °C. The abundance of RhTMBIM1-Flag protein at different time points was determined by immunoblotting using an anti‐Flag antibody. Actin was used as an internal control.

**Figure S11. RhTMBIM7 negatively regulates petal senescence.**

A) Senescence phenotype of control and *RhTMBIM7*-silenced flowers. Bars, 5 cm.

B) Flower longevity of control and *RhTMBIM7*-silenced flowers. Data are shown as means ± SD (n=10).

C) *RhTMBIM7* transcript level in control and *RhTMBIM7*-silenced petals.

D) Expression of senescence marker gene *RhSAG12* in control and *RhTMBIM7*-silenced petals.

*RhUBI2* was used as a reference gene. Data are shown as means ± SD, n=5 in C) and D). Asterisks indicate statistically significant differences as determined by *t*-test (**P<0.01, ****P<0.0001).

**Figure S12. RhSAREL accelerates petal senescence by destabilizing RhTMBIM7.**

A) Senescence phenotype of TRV control, TRV-*RhSAREL* and TRV-*RhSAREL*/*RhTMBIM7* flowers. Bars, 5 cm.

B) Flower longevity of TRV control, TRV-*RhSAREL* and TRV-*RhSAREL*/*RhTMBIM7* flowers. Data are shown as means ± SD (n=10).

C, D) Expression of *RhSAREL* (C) and *RhTMBIM7* (D) in TRV control, TRV-*RhSAREL* and TRV-*RhSAREL*/*RhTMBIM7* petals.

E) Expression of senescence marker gene *RhSAG12* in TRV control, TRV-*RhSAREL* and TRV-*RhSAREL*/*RhTMBIM7* petals.

*RhUBI2* was used as a reference gene. Data are shown as means ± SD, n=5 in C)–E). Different letters indicate statistically significant differences (P＜0.05) analyzed by one-way ANOVA with Tukey’s test.

**Figure S13. RhSAREL blocks the Ca^2+^ leakage channel of RhTMBIM1.**

A) Protein structure alignment of RhTMBIM1 and BsYetJ. The 3D structure of RhTMBIM1 was predicted by ColabFold (https://github.com/sokrypton/ColabFold). The alignment was performed using RCSB (https://www.rcsb.org/alignment). The template modeling score (TM-score) is an index of protein structural similarity, wherein a TM-score greater than 0.5 indicates that two proteins likely share the same fold.

B, C, D) The docking results of RhSAREL_1–49_ and RhTMBIM1. Docking between RhSAREL_1–49_ (marine blue) and RhTMBIM1 (wheat yellow) was conducted using three web servers: ClusPro (B), ZDOCK (C), and HDOCK (D). The corresponding websites are as follows: ClusPro (https://cluspro.org/home.php), ZDOCK (https://zdock.wenglab.org), and HDOCK (http://hdock.phys.hust.edu.cn).

E) Schematic diagrams of the different expression cassettes in the Super1300 vector for callus transformation. 2A peptides were used to link different genes in a single open reading frame, enabling the production of multiple proteins via self-cleavage. RB, right border; LB, left border.

**Figure S14. RhTMBIM1 inhibits cell death during petal senescence.**

A, B) Trypan blue staining of rose petals with transient silencing (A) or overexpression (B) of *RhTMBIM1*. Bars, 1 cm.

C, D) Quantitative measurement of ion leakages from rose petals with transient silencing (C) or overexpression (D) of *RhTMBIM1*.

E, F) Expression of PCD-associated genes in petals with transient silencing (E) or overexpression (F) of *RhTMBIM1*.

The outermost petals of infected flowers at stage 5 were sampled for phenotypic and gene expression analysis. *RhUBI2* was used as a reference gene. Data are shown as means ± SD, n=3 in C)–F). Asterisks indicate statistically significant differences as determined by two-way ANOVA with Sidak’s test (**P<0.01, ***P<0.001).

**Figure S15. Partial sequence alignment of TMBIM proteins from rose and human.**

The red arrowheads indicate aspartate residues critical for the function of TMBIM proteins. The protein sequences used are listed below: RhTMBIM1, XP_024170505.1; RhTMBIM2, XP_040375241.1; RhTMBIM3, XP_024186666.1; RhTMBIM4, XP_040364523.1; RhTMBIM5, XP_024168153.1; RhTMBIM6, XP_024168153.1; RhTMBIM7, XP_024196807.1; RhTMBIM8, XP_024161613.1; HsTMBIM1, NP_071435.2; HsTMBIM2, NP_036438.2; HsTMBIM3, NP_000828.1; HsTMBIM4, NP_057140.2; HsTMBIM5, NP_055209.2; HsTMBIM6, NP_003208.2. Rh, *Rosa chinensis*; Hs, *Homo sapiens*.

**Figure S16. Protein structure alignments of RhTMBIM7 with BsYetJ and RhTMBIM1.**

The 3D structure of RhTMBIM7 was predicted by ColabFold (https://github.com/sokrypton/ColabFold). The alignments were performed using RCSB (https://www.rcsb.org/alignment). The template modeling score (TM-score) is an index of protein structural similarity.

**Figure S17. RhTMBIM1 and RhTMBIM7 are involved in the maintenance of Ca²⁺ homeostasis.**

A) Time-course analysis of cytosolic Ca²⁺ dynamics in EV, RhTMBIM1/GFP, and RhTMBIM7/GFP callus lines after treatment with 5 mM H_2_O_2_. The luminescence signal was acquired at 1-second intervals.

B) Quantification of the highest cytosolic Ca^2+^ concentration in A).

Data are shown as means ± SD, n=5 in A) and B). Different letters indicate statistically significant differences (*P*<0.05) analyzed by one-way ANOVA with Tukey’s test.

**Figure S18. Sequence alignment of proteins homologous to RhTMBIM1 and RhTMBIM7 from different species.**

The lysine residues are highlighted in red. Filled black triangles indicate conserved lysine residues in TMBIM1 homologous proteins; open black triangles represent conserved lysine residues in TMBIM7 homologous proteins; open red triangles denote lysine residues conserved in both TMBIM1 and TMBIM7 homologous proteins. The protein sequences used are listed below: RhTMBIM1, *Rosa hybrida* XP_024170505.1; AtBI-1, *Arabidopsis thiliana* NP_199523.1; OsBI-1, *Oryza sativa* NP_001393352.1; PpBI-1, *Physcomitrium patens* XP_024384587.1; RhTMBIM7, *Rosa hybrida* XP_024196807.1; AtLFG, *Arabidopsis thiliana* NP_567466.1; OsBI1-like, *Oryza sativa* XP_015640550.1; PpBI1-like, *Physcomitrium patens* XP_024371886.1.
